# The *Pah-R261Q* mouse reveals oxidative stress associated with amyloid-like hepatic aggregation of mutant phenylalanine hydroxylase

**DOI:** 10.1038/s41467-021-22107-1

**Published:** 2021-04-06

**Authors:** Oscar Aubi, Karina S. Prestegård, Kunwar Jung-KC, Tie-Jun Sten Shi, Ming Ying, Ann Kari Grindheim, Tanja Scherer, Arve Ulvik, Adrian McCann, Endy Spriet, Beat Thöny, Aurora Martinez

**Affiliations:** 1 Department of Biomedicine, University of Bergen, Bergen, Norway; 2 Division of Metabolism, University Children’s Hospital Zürich and Children’s Research Centre, Zürich, Switzerland; 3 Bevital AS, Laboratoriebygget, Bergen, Norway

**Keywords:** Metabolic disorders, Experimental models of disease

## Abstract

Phenylketonuria (PKU) is caused by autosomal recessive variants in phenylalanine hydroxylase (*PAH*), leading to systemic accumulation of L-phenylalanine (L-Phe) that may reach neurotoxic levels. A homozygous *Pah*-*R261Q* mouse, with a highly prevalent misfolding variant in humans, reveals the expected hepatic PAH activity decrease, systemic L-Phe increase, L-tyrosine and L-tryptophan decrease, and tetrahydrobiopterin-responsive hyperphenylalaninemia. *Pah*-*R261Q* mice also present unexpected traits, including altered lipid metabolism, reduction of liver tetrahydrobiopterin content, and a metabolic profile indicative of oxidative stress. *Pah-R261Q* hepatic tissue exhibits large ubiquitin-positive, amyloid-like oligomeric aggregates of mutant PAH that colocalize with selective autophagy markers. Together, these findings reveal that PKU, customarily considered a loss-of-function disorder, can also have toxic gain-of-function contribution from protein misfolding and aggregation. The proteostasis defect and concomitant oxidative stress may explain the prevalence of comorbid conditions in adult PKU patients, placing this mouse model in an advantageous position for the discovery of mutation-specific biomarkers and therapies.

## Abstract (web summary)

Phenylketonuria (PKU) is caused by autosomal recessive variants in phenylalanine hydroxylase (PAH) and can lead to neurotoxicity. Here the authors describe a mouse model of PKU based on a mutation in phenylalanine hydroxylase (R261Q) which replicates traits of this disease and shows a proteostasis defect and oxidative stress, implying a gain-of-function contribution to the disease phenotype.

## Introduction

Phenylketonuria (PKU; MIM261600) is an autosomal recessive inborn error of metabolism characterized by the inability to break down the amino acid L-phenylalanine (L-Phe). PKU is primarily caused by mutations in the human *PAH* gene (NM_000277.2) encoding phenylalanine hydroxylase (PAH; EC 1.14.16.1). PAH is a tetrameric, non-heme iron aromatic amino acid hydroxylase that catalyzes the hydroxylation of L-Phe to L-tyrosine (L-Tyr) using molecular oxygen as additional substrate and the cofactor (6*R*)-5,6,7,8-tetrahydrobiopterin (BH_4_)^1,2^. This is the rate-limiting step in the catabolic degradation of L-Phe, which occurs predominantly in the cytoplasm of hepatic cells. A consequence of deficient PAH catalysis is the accumulation of L-Phe in the blood and ultimately in the brain of untreated patients, causing growth retardation, intellectual disability, and behavioral and neuropsychiatric disorders^3^. PKU has a prevalence of approximately 1:10,000 livebirths worldwide and can be classified, based on the off-diet blood L-Phe concentrations, as mild hyperphenylalaninemia (HPA) (120–600 µmol/L), mild PKU (600–1200 µmol/L), and classic PKU (>1200 µmol/L)^3^. The low-Phe diet is the cornerstone of PKU/HPA management and prevents the most severe consequences of the disease. However, controlled studies have shown that early treated PKU patients present several psychiatric disturbances as adults, notably depression and anxiety-related disorders^4^. Moreover, recent investigations have revealed an elevated risk of comorbidities with unexplained etiology in both early- and late-treated adult PKU patients, with a high prevalence of cardiovascular and renal diseases and overweight^5,6^. In the last years, many new treatments for PKU have been approved or are in clinical development^7^. A fraction of patients typically with mild and moderate *PAH* mutations are responsive to synthetic formulations of BH_4_ (Sapropterin, Kuvan®), which is often used in combination with a less restrictive diet^8^. Notwithstanding the considerable amount of accumulated knowledge on PKU, there is a need for a more profound mechanistic and pathophysiological understanding of the disease, as well as novel therapies. These required studies would greatly benefit from the availability of useful model organisms.

Mouse (*Mus musculus*) models are a powerful research tool owing to the small size, high reproductive rate, and relative ease of genetic manipulation, compared to other mammals, and are therefore most commonly selected to study human disease^9^. There are evident differences between mice and humans, primarily related to evolutionary divergences, for instance in size, metabolic rate, life expectancy, and immune system, but overall, the genetic and physiological similarities are high^9^. The first generation of mouse models of PKU were created by phenotype-driven N-ethyl-N-nitrosourea (*Enu*) germline mutagenesis. In this manner, three HPA/PKU mouse models have previously been established; namely, (i) *Enu1* (*enu*^1^ allele), with the p.V106A-PAH mutation, located in the PAH regulatory domain^10^; (ii) *Enu2* (*enu*^2^ allele), with the p.F263S-PAH mutation, located in the catalytic domain^10^; and (iii) *Enu3* (*enu*^3^ allele), with a splice site mutation generating frameshifted amino acids and premature termination codon^11^. *Enu2* and *Enu3* mice exhibit high blood L-Phe concentrations (>1200 µmol/L) and appear as suitable models for severe, classic PKU, with a total absence of PAH activity albeit normal protein stability (*Enu2*), or total absence of expressed PAH protein and activity (*Enu3*)^11^. In contrast, *Enu1* mice present reduced PAH stability and thus decreased steady-state levels of PAH protein and enzymatic activity (approximately 5% of normal controls), leading to mild HPA^12^. The available mouse models have undoubtedly contributed to a better understanding of PKU at a biochemical and behavioral level and have allowed testing of novel therapies such as enzyme substitution^13^ or genome base editing^14^. An increasing body of evidence indicates that PKU is a prototypic genetic conformational disorder wherein the principal pathogenic determinant is the degree of PAH protein instability caused by the specific mutations^15^. The available strains do not adequately represent this primary pathogenic mechanism (*Enu2* and *Enu3*) or include murine mutations that are non-existent or low recurrent in the human *PAH* gene (*Enu1*), prompting us to generate a PKU mouse model with a common *PAH* mutant associated with protein misfolding.

There are over 1100 registered human PAH variants (http://www.biopku.org/), among which the nucleotide aberration c.782 G > A in *Pah* exon 7 coding for p.Arg261Gln (p.R261Q) mutation seems to be an optimal candidate to generate a knock-in mouse model. The *R261Q* mutation is one of the most abundant among PKU patients, with an average allele frequency of approx. 6% (9-14% in Mediterranean countries and the Middle East) and ~2% of patients homozygous for this mutation (up to 12% in Mediterranean countries and the Middle East)^15,16^ (http://www.biopku.org/). The associated phenotype when in homozygosity, exhibits an unusual and unexplained variability from mild PKU to classic PKU, with approximately 78% of the patients being responsive to BH_4_^15–17^ (http://www.biopku.org/). The *R261Q* mutation has been predicted^15^ and indeed proven to result in unstable and misfolded PAH^18,19^.

Hence, in the reported custom-made mouse model, the mutation c.782G > A was introduced in the *Pah* gene by CRISPR/Cas9 technology based on the use of programmable nucleases as a tool for targeted gene-editing, which is an efficacious and precise genome engineering method^20^. In this work, we present the generation and metabolic, biochemical and biological characterization of this *Pah-R261Q* knock-in mouse line. The results obtained highlight (i) the robustness of this mouse model as a general archetype for mild HPA associated with PAH instability and misfolding, and (ii) the observation of large amyloid-like aggregates of mutant (p.R261Q-PAH) in vivo, which appears associated to the observed proteostasis dysregulation, oxidative stress and additional comorbidities. Overall, the *Pah-R261Q* mouse model paves the way for new exploratory avenues of research and treatment.

## Results

### Generation, genotyping, and breeding of *Pah-R261Q* mice

There is a high PAH sequence homology (92.5% identity) between mouse and human PAHs, with Arg261 being in an evolutionarily conserved region (Supplementary Fig. 1a). Structurally, the residue Arg261 establishes several intra- and inter-subunit H-bonding and electrostatic contacts in the dimers that are crucial to maintaining the stability of the protein as well as proper oligomeric configuration^21,22^ (detailed in Supplementary Fig. 1b, c). The mutation p.R261Q is thus expected to trigger disruption of this interaction network and, as seen in expression analyses in different systems, elicit an unstable protein^18,19,23^ without substantially affecting the catalytic efficiency of the folded tetramer^19^.

The custom-made mouse model with the p.R261Q-PAH mutation was generated by CRISPR/Cas9 genome editing technology, as schematically represented in Supplementary Fig. 2a, and proven to have the correct genotype (Supplementary Fig. 2b, c). The designed primers for genotyping amplified a 537 bp polymerase chain reaction (PCR) product, which, after restriction fragment analysis with endonuclease BsmI, made it possible to discriminate electrophoretically between *Pah*^*WT/WT*^ (2 fragments: 294 and 243 bp), homozygous *Pah*^*R261Q/R261Q*^ (3 fragments: 243, 171, and 123 bp), and heterozygous *Pah*^*R261Q/WT*^ (4 fragments: 294, 243, 171, and 123 bp) mice (Supplementary Fig. 2c). There is evidence that BLAST hits with three or more total nucleotide mismatches have a low probability of off-target effects, specifically if two of these mismatches are situated in the seed region^24^. In any case, we evaluated all 16 candidate loci susceptible to being secondarily affected by the guide RNA sequence (Supplementary Table 1). These genes were subjected to heteroduplex analysis, all showing wild-type sequence, with no detection of off-target interactions.

A retrospective examination of the maternal genotype effect in the breeding revealed no abnormalities between homozygous *Pah*^*R261Q/R261Q*^ mice (referred to as *Pah-R261Q*) and *Pah*^*WT/WT*^ (*WT*) mice with respect to litters per mated female (3.7 ± 0.6 vs. 4.0) and progeny per litter (7.1 ± 0.4 vs. 7.0)^25^. Furthermore, the compilation of historical data confirmed the expected offspring genotypic distribution as predicted by Mendelian laws.

### *Pah-R261Q* mice exhibit mild HPA, higher body weight in the case of males, and reduced respiratory exchange ratio

**Fig. 1:**
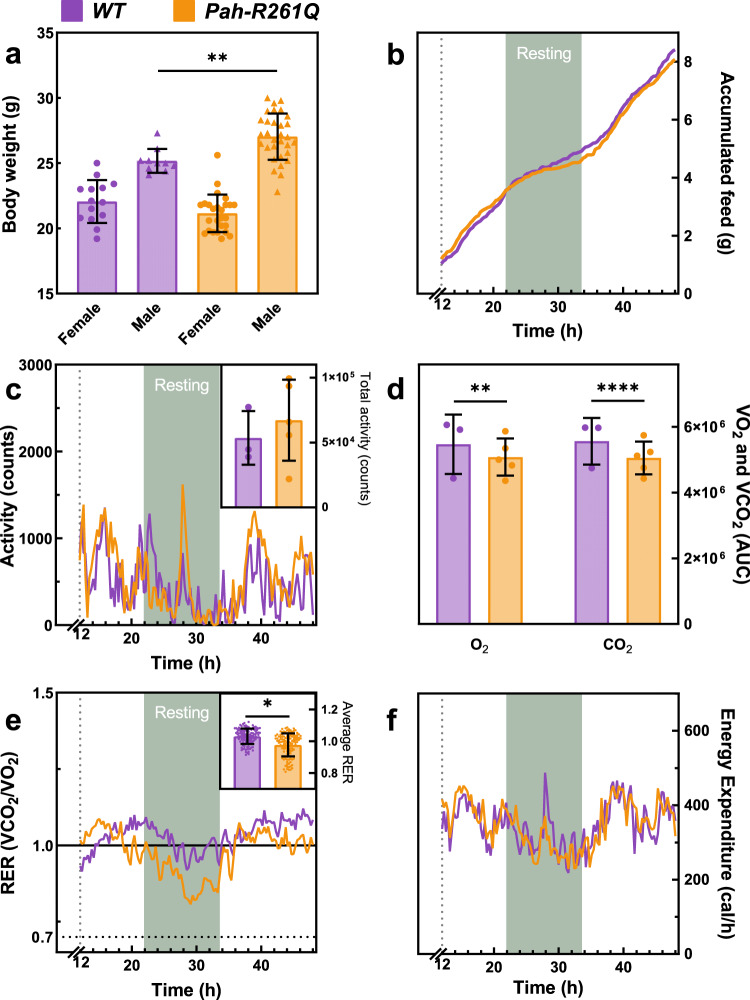
Physiological and metabolic characterization of *Pah-R261Q* compared with *WT* mice. **a** Bodyweight distribution by sex and genotype. The weight of *WT* mice (controls) was in agreement with averaged registered data (https://www.jax.org/strain/000664). Data are presented as mean ± SD, with individual values plotted as circles (females) and triangles (males) (*n* = 10 *WT* male, 14 *WT* female, 31 *Pah-R261Q* male, 26 *Pah-R261Q* female mice). Statistical significance for the weight difference for males in the two groups was calculated by two-tailed unpaired *t* test; *p* = 0.0031 (**). **b**–**f** Metabolic cage experiments, performed for 48 h, with 12 h of acclimation followed by 36 h of recordings. *n* = 3 *WT* and 5 *Pah-R261Q* mice in independent experiments, with one mouse per cage and 121 observations/animal. **b** Cumulative feed consumption (g). **c** Mice activity with continuous recording, expressed as mean ± SD. Inset, total activity for each mouse group presented as mean ± SD, individual values are plotted as circles. **d** Total Volume of O_2_ consumed and volume of CO_2_ produced for each mice type, obtained from the integration of the area under the curve (AUC) from data in Supplementary Fig. 3. Data are presented as the mean AUC ± SD, with individual values plotted as circles. Statistical significance for the difference between both mice groups was calculated by two-tailed unpaired *t* test; *p* = 0.0011 (**) for O_2_ and *p* < 0.0001 (****) for CO_2_. **e** Respiratory exchange ratio (RER) along the recording time. Inset: averaged RER presented as mean ± SD; the circles represent mean for the group at each time point. Statistical significance for differences between both groups was calculated by two-tailed unpaired *t* test; *p* < 0.0001 (****). **f** Energy expenditure obtained by indirect calorimetry expressed as mean ± SD. In all panels, the data for *WT* are depicted in purple and *Pah-R261Q* in ochre. Source data are provided as a Source Data file.

*Pah-R261Q* mice presented a small but significant increase in basal blood L-Phe levels, measured in dried blood spots in 3-month old mice (108.0 ± 36.6 µM*, n* = 23 mice) compared to both *WT* (59.9 ± 7.7 µM, *n* = 9; *p* < 0.0001) and heterozygous *Pah*^*R261Q/WT*^ (71.22 ± 21.86 µM; *n* = 6; *p* = 0.0201), analyzed by Brown–Forsythe and Welch ANOVA test followed by Dunnett’s multiple comparisons tests. Source data are provided as a Source Data file. The blood L-Phe level in *Pah-R261Q* corresponds to very mild HPA in human subjects. In contrast to the *Enu2* PKU mouse model, which presents weight and length reduction, hypopigmentation, behavioral, and neurological problems^26^, *Pah-R261Q* were no different from their heterozygote and *WT* counterparts in length, pigmentation, and behavior. Three-month-old male *Pah-R261Q* mice, however, were weightier than their *WT* counterparts (27.8 ± 0.4 vs. 25.1 ± 0.3 g, respectively) (Fig. 1a). *Pah-R261Q* females were as expected lighter than males and were not different in weight than their *WT* counterparts (22.1 ± 1.6 vs. 21.1 ± 1.4 g, respectively). Apart from the higher body weight in mutant males, we did not find gender-associated variations for any other parameter or metabolite measured in this work; thus, the mice groups for each experiment included evenly distributed males and females.

Various physiological murine parameters were controlled for the 48 h metabolic cage examinations for *WT* and *Pah-R261Q* (12 h acclimatization and 36 h of measured observations). The amount of food intake was equal (Fig. 1b), and no significant changes in activity and movement patterns were identified (Fig. 1c). However, the rates of O_2_ consumption and CO_2_ production normalized to body mass (VO_2_ and VCO_2_, respectively) were both decreased for *Pah-R261Q* compared with *WT* (Fig. 1d and Supplementary Fig. 3a, b), and the calculated respiratory exchange ratio (RER = VCO_2_/VO_2_) was also slightly lower for *Pah-R261Q* than for *WT* (0.988 ± 0.087 vs. 1.014 ± 0.093; the average for the total 36 h experimentation) (Fig. 1e, inset). RER values are approximately 1.0, 0.8, and 0.7 for carbohydrates, proteins, and fat, respectively, as sole metabolic fuel^27^. Nevertheless, in heavy activity periods RER increases and reaches values >1^28^, and RER values reflect metabolic fuel utilization more accurately during periods of rest or mild exercise^29^. During the 12 h resting period, RER was closer to 0.8 for *Pah-R261Q* and 1 for *WT* mice (Fig. 1e), indicating a higher utilization of fat and protein as a fuel source during this period among the mutant mice^30^. The decreased RER at rest also contributed to lower energy expenditure in the same period compared with *WT* mice, even for non-weight-normalized values (Fig. 1f), although this difference did not translate into significantly lower energy expenditure for the mutant mice per day.

### Metabolic characterization of *Pah-R261Q* mice show lipid metabolism alterations and oxidative stress

Detailed metabolic profiling of *Pah-R261Q* compared with *WT* mice was performed by measuring 72 relevant metabolic biomarkers in extracted blood serum samples from 4-month-old mice. The complete list of metabolites and the results obtained are presented in Supplementary Table 2. Table 1 summarizes the individual values for the 17 metabolites displaying differences at *p* < 0.1 level, with either higher or lower blood serum concentrations for *Pah-R261Q* compared to *WT*. This *p* value was selected to avoid type II error due to the limited sample size.

**Table 1:** Blood serum concentrations of the metabolites whose levels were increased/decreased in *Pah-R261Q* mice in respect to the control *WT* group. Concentrations are expressed as arithmetic mean ± SD; *n* = 19 *WT* and 19 *Pah-R261Q* mice.

Metabolite (name)	*WT* (µM)	*Pah-R261Q* (µM)	Difference	*p* Value (MW)^a^
Phenylalanine	71.9 (10.3)	113 (22)	41.1	0.0000004
β-Hydroxybutyrate	150 (99)	282 (133)	132	0.029
Trimethyllysine	0.803 (0.165)	0.976 (0.213)	0.173	0.050
Leucine	143 (23)	164 (42)	21	0.075
Isoleucine	87.5 (12.8)	101 (24)	13.5	0.091
α-Ketoglutaric acid	38.6 (15.2)	26.3 (10.8)	−12.3	0.003
Glutamic acid	39.5 (20.7)	29.4 (10.2)	−10.1	0.008
Alanine	444 (71)	362 (80)	−82	0.010
Tryptophan	103 (24)	82.9 (30.9)	−20.1	0.013
Quinolinic acid	0.178 (0.090)	0.130 (0.041)	−0.48	0.023
Creatine	154 (40)	127 (30)	−27	0.026
Aspartic acid	27.4 (14.4)	22.7 (11.0)	−4.7	0.043
Glutamine	687 (76)	622 (112)	−65	0.043
Tyrosine	81.3 (25.3)	77.9 (14.0)	−3.4	0.050
Methylmalonic acid	0.701 (0.095)	0.585 (0.118)	−0.116	0.060
Kynurenine	0.740 (0.234)	0.573 (0.264)	−0.167	0.080
Proline	90.2 (25.4)	73.0 (17.2)	−17.2	0.085

^a^Two-tailed *p* values for differences between serum concentration in *WT* and *Pah-R261Q* mice were obtained from Mann Whitney (MW) *U* test.

See also Supplementary Table 2.

A blood serum L-Phe concentration in the mutant mice corresponding to very mild HPA (113 ± 22 µM vs. 71.9 ± 10.3 µM for *WT*) was obtained in this study, similar to the values obtained from dried blood spots (see above). As seen in Table 1, the increased serum L-Phe was accompanied by decreased levels of L-Trp and L-Tyr, markers of the HPA phenotype, as well as decreased quinolinic acid and a trend for reduced kynurenine, both downstream metabolites of L-Trp. Interestingly, serum trimethyllysine, leucine, and isoleucine, which have been shown to increase in adiposity and altered lipid metabolism in humans^31^, were elevated in *Pah-R261Q* (Table 1). Moreover, increased β-hydroxybutyrate is also an established biomarker associated with impaired glucose homeostasis, diabetes, and defense against oxidative stress^32,33^. Also, other serum metabolites observed in lower concentrations in *Pah-R261Q* have previously been linked to oxidative stress and immune function, such as α-ketoglutaric acid, glutamic acid, and quinolinic acid^34–37^. These biomarkers are tightly associated metabolically to creatine and methylmalonic acid, and to the amino acids aspartic acid, alanine, glutamine, and proline, all with decreased trends (Table 1).

The incorporation of these metabolites to the Krebs cycle—a central hub of metabolism—through anaplerotic reactions is increased in situations of oxidative and cellular stress^38^. Furthermore, the reduction in anaplerotic metabolites and increase in β-oxidation in *Pah-R261Q* are in agreement with increased utilization of proteins and fats as an energy source, as also inferred by the lower resting-state RER in these mice compared to *WT* (Fig. 1e). We acknowledge that the use of a high, explorative *p* value cutoff (*p* < 0.1) may have generated spurious hits among the metabolite biomarkers. However, we believe that the approach is justified by the overall coherence of the findings that support lipidic metabolic alterations and oxidative stress in the *Pah-R261Q* mice, in addition to the expected mild HPA.

### *Pah-R261Q* mice show no apparent neurological alteration but a remarkable decrease of hepatic BH_4_

**Fig. 2:**
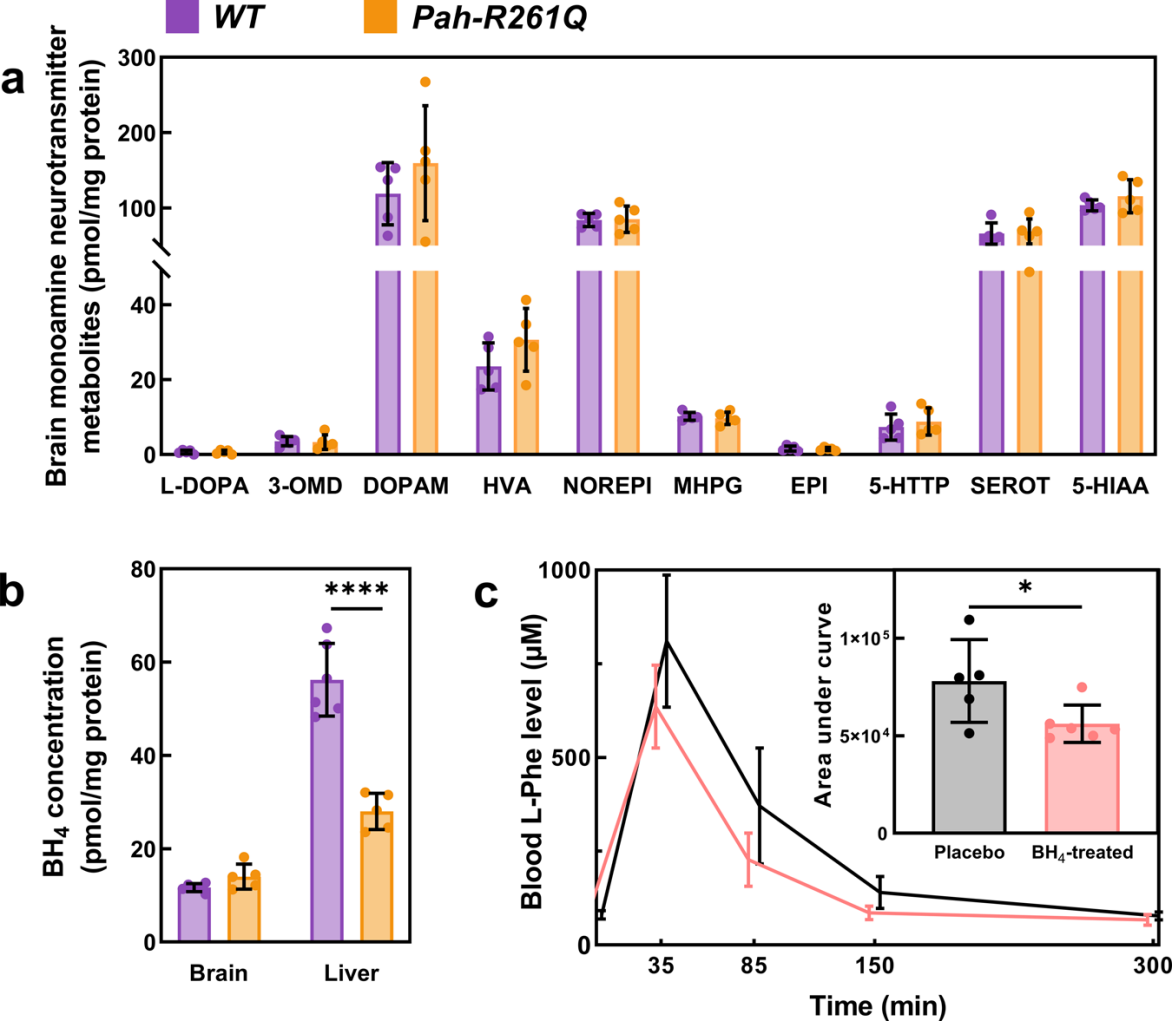
Neurotransmitter and BH_4_ content, and BH_4_-responsiveness in the *Pah-R261Q* mouse model. **a** Monoamine neurotransmitter content in brain lysates; data are presented as mean ± SD, individual values are plotted as circles (*n* = 5 *WT* and 5 *Pah-R261Q* mice). Abbreviations (from left to right): levodopa, 3-ortho-methyldopa, dopamine, homovanillic acid, norepinephrine, 3-methyl-4-hydroxyphenylglycol, epinephrine, 5-hydroxytryptophan, serotonin, and 5-hydroxyindoleacetic acid. **b** BH_4_ determination in whole brain and liver lysates, presented as mean ± SD, individual values are plotted as circles (*n* = 6 *WT* and 5 *Pah-R261Q*). Statistical significance for the difference in brain BH_4_ content between both groups was calculated by two-tailed unpaired *t* test; *p* < 0.0001 (****). **c** Blood L-Phe concentration after L-Phe challenge in placebo-control (black) and BH_4_-treated (pink) *Pah-R261Q* mice (*n* = 5 placebo and 6 treated mice). L-Phe (200 µg L-Phe/g body weight) was administered by i.p. at time 0 and L-Phe concentration was monitored at 0, 35, 90, 150, and 300 min. The BH_4_ treated mice received (by i.p.) 20 mg/kg BH_4_ in 2% ascorbic acid and 10% DMSO, for 4 days, twice a day, previous to L-Phe administration, and the placebo control received the same solution without BH_4_. Data are presented as mean ± SD. Inset, area under the curve (AUC) for the time dependence of L-Phe concentration between 0 and 300 min for placebo and BH_4_-treated groups. Individual values are represented by circles. Statistical significance for the difference between both groups was calculated by two-tailed unpaired *t* test; *p* = 0.0299 (*). In panels **a** and **b** the data for *WT* are depicted in purple and for *Pah-R261Q* in ochre. Source data are provided as a Source Data file.

The elapsed time for a mouse to maintain its balance on a rotating rod is a good indicator of possible neurological deficits, as shown for the *Enu2* mouse^39^. As illustrated in Supplementary Fig. 3c, *Pah-R261Q* had comparable performance to *WT* on the rotarod test, supporting that the *PAH* mutation has no impact in neuromuscular function or motor coordination, at least for young mice. We also corroborated no alterations in the levels of aromatic amino acids L-Phe, L-Tyr, and L-Trp (Supplementary Table 3) and monoamine neurotransmitters (Fig. 2a) in the brain. In addition, as seen in Fig. 2, we confirmed no significant difference in the total levels of BH_4_ in the brain, where BH_4_ acts as a cofactor of the other aromatic amino acid hydroxylases tyrosine hydroxylase (TH) and tryptophan hydroxylase 2 (TPH2) and of neuronal nitric oxide synthase (NOS)^2^. However, the concentration of BH_4_ in the liver, where it acts as the essential cofactor for the hydroxylating PAH reaction, showed a startling 50% reduction in *Pah-R261Q* (28.0 ± 1.7 pmol BH_4_/mg protein) compared with *WT* mice (56.2 ± 3.2 pmol/mg) (Fig. 2b).

### *Pah-R261Q* mice are sensitive to L-Phe challenge concomitant with BH_4_ responsive hyperphenylalaninemia

When we administered *Pah-R261Q* mice an L-Phe challenge—equivalent to the L-Phe loading test that is applied to HPA/PKU patients for their phenotypic classification^40^—a transient but very prominent elevation of blood L-Phe values was observed. As shown in Supplementary Fig. 4, 40 min after i.p. injection with 200 µg L-Phe/g body weight, *Pah-R261Q* mice presented a massive increase in L-Phe concentrations (990 ± 220 µM), before returning to basal levels ca. 300 min later. A similar L-Phe challenge caused much lower increases of L-Phe concentrations in heterozygous and *WT* mice (Supplementary Fig. 4). The transient L-Phe-increase in *Pah-R261Q* mice allowed us to investigate the response to BH_4_-treatment. As seen in Fig. 2c, significantly lower blood L-Phe level was measured after an L-Phe challenge (200 µg/g body weight) was administered following a BH_4_ treatment (20 mg/kg body weight for 4 days, twice a day^41^), compared with placebo. BH_4_ treatment resulted in a 28% decrease in L-Phe content as calculated from the area under the curve (Fig. 2c, inset). Patients with the *R261Q* mutation in homozygosity present variable HPA phenotype but about 74% are positive responders to BH_4_ treatment (http://www.biopku.org/), and the results with the mutant mice are consistent with the BH_4_-responsive phenotype.

### Hepatic p.PAH-R261Q protein presents increased ubiquitination and aggregation

**Fig. 3:**
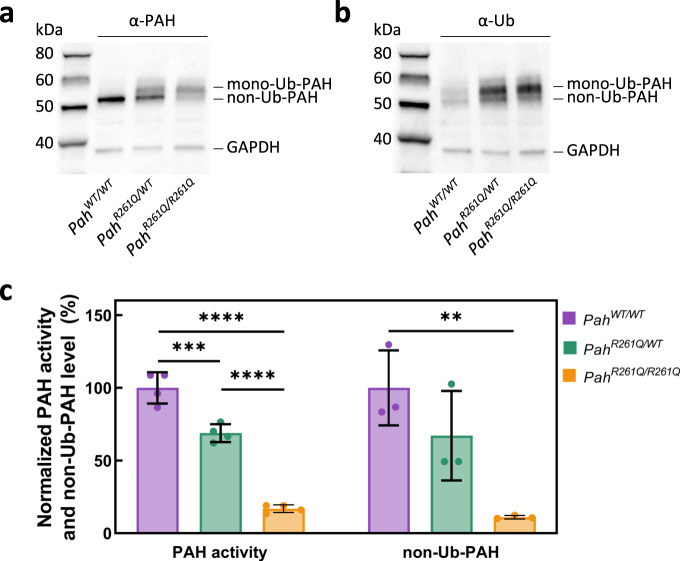
PAH content in liver lysates of homozygous and heterozygous *Pah-R261Q mice*. **a** Western blots for immunodetection of PAH (α-PAH) (**a**) and ubiquitinated protein (α-Ub) (**b**) showing the decrease in non-ubiquitinated PAH (non-Ub-PAH; ~51 kDa band) and increase of mono-ubiquitinated PAH (mono-Ub-PAH; ~56 kDa) from genotype *Pah*^*WT/WT*^ to *Pah*^*R261Q/WT*^ to *Pah*^*R261Q/R261Q*^. The blots are representative from *n* = 3 replicates for each mice group. GAPDH was used as loading control. **c** Overview of relative PAH specific activity normalized to activity in *Pah*^*WT/WT*^ liver lysates (23.2 ± 2.4 nmol L-Tyr/min/mg protein) (*n* = 4 mice for each genotype) and non-Ub-PAH protein (51 kDa) levels from densitometric analysis normalized to both *Pah*^*WT/WT*^ liver lysates as well as to GAPDH loading control (*n* = 3 mice for each genotype). Data are presented as mean ± SD for *Pah*^*WT/WT*^ (purple), *Pah*^*R261Q/WT*^ (green), and *Pah*^*R261Q/R261Q*^ (ochre), individual values are represented as circles. Differences between genotypes were analyzed by one-way ANOVA followed by Tukey test; differences in PAH activity, *p* = 0.0005 (***) for *Pah*^*WT/WT*^ vs. *Pah*^*R261Q/WT*^, *p* < 0.0001 (****) for both *Pah*^*WT/WT*^ vs. *Pah*^*R261Q/R261Q*^ and *Pah*^*R261Q/WT*^ vs. *Pah*^*R261Q/R261Q*^; differences in PAH level, *p* = 0.0080 (**) for *Pah*^*WT/WT*^ vs*. Pah*^*R261Q/R261Q*^. Source data are provided as a Source Data file.

After the investigation of the metabolic status of *Pah-R261Q* mice, we also studied the effect of the mutation on the function and stability of PAH in the mouse liver. Immunodetection by Western blot of the PAH protein (p.R261-PAH and WT-PAH, for the mutant and WT proteins, respectively) in liver lysates showed the typical 51-kDa PAH band for *WT* mice, and two PAH bands at 51- and 56-kDa for the heterozygous *Pah*^*R261Q/WT*^ and homozygous *Pah*^*R261Q/R261Q*^ (*Pah-R261Q*) mutant mice (Fig. 3a). The 56-kDa PAH band, present in both mice with the mutant allele, was recognized by the anti-ubiquitin antibody (Fig. 3b). This band has been identified as mono-ubiquitinated PAH in previous studies with *Enu1* and *Enu1/2* HPA mouse models^42,43^. The 51-kDa non-ubiquitinated PAH band was strongly reduced in *Pah-R261Q*, as best observed in the immunoquantified PAH levels normalized to *WT* control mice (Fig. 3c). We also measured PAH activity in the liver lysates, and for each genotype, the relative specific activity correlated well with the relative levels of non-ubiquitinated PAH protein in liver lysates (Fig. 3c). Nevertheless, the results with *Pah-R261Q* (11.6 ± 1.5% non-ubiquitinated protein vs. 16.9 ± 3.3% specific activity, both relative to *WT*) support low level of PAH activity for the ubiquitinated enzyme.

**Fig. 4:**
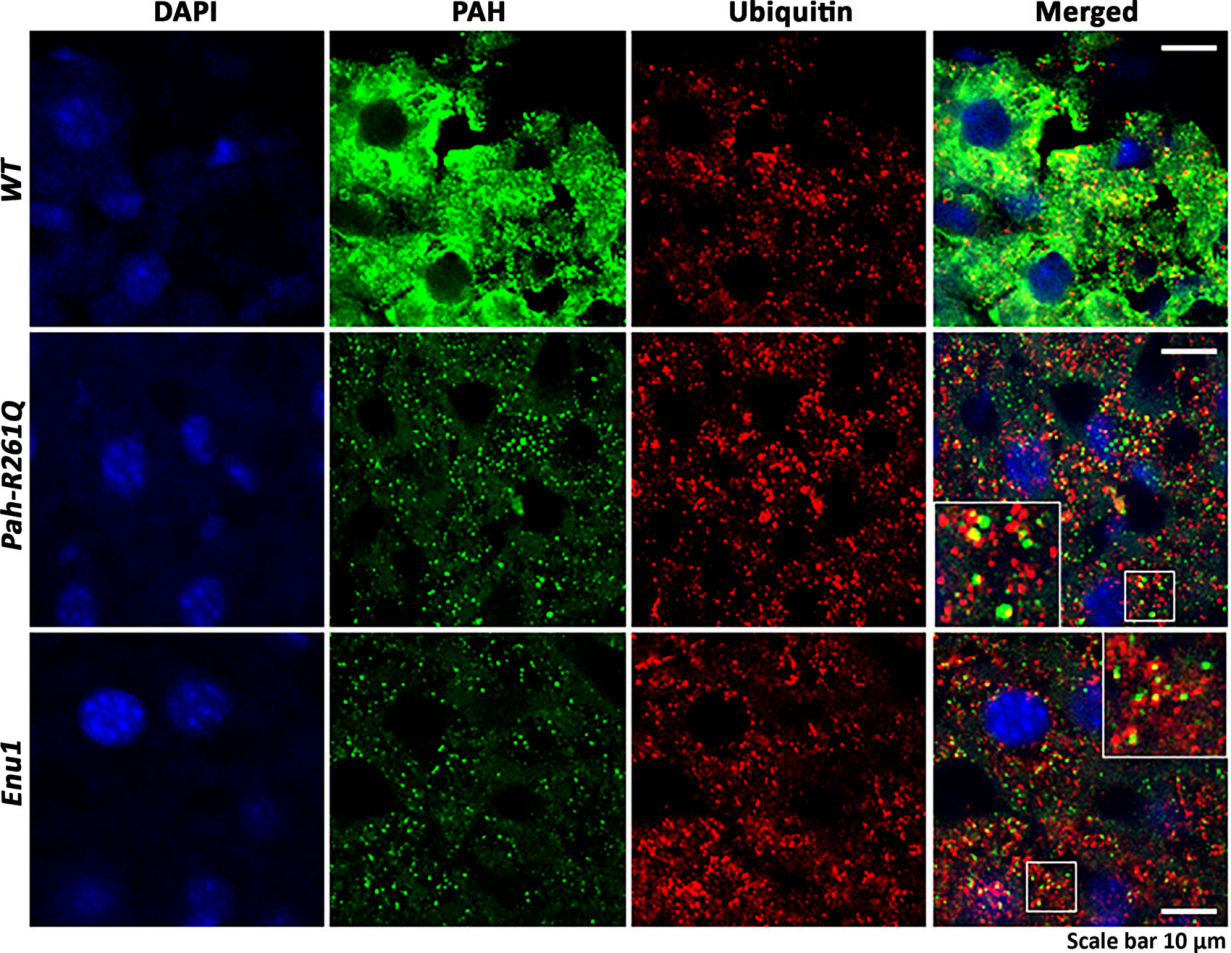
Distribution of PAH in hepatic tissue of *WT* and mouse models *Pah-R261Q* and *Enu1*. Immunofluorescence of PAH and ubiquitin (Ub) detection in hepatic tissue of *WT, Pah-R261Q,* and *Enu1* mice, revealing the distribution pattern of PAH (green) and Ub (red). PAH was strongly reduced in both *Enu1* and *Pah-R261Q* when compared to *WT*, whereas Ub was highly expressed in both mutant mice. The micrographs are representative for *n* = 3 biological replicates in each mice group. The fluorescence intensity (mean ± SD) calculated in 14 stacks of confocal images, relative to *WT* (=1), was 0.264 ± 0.105 (*Pah-R261Q*) and 0.154 ± 0.029 (*Enu1*) for PAH, and 1.315 ± 0.035 (*Pah-R261Q*) and 1.405 ± 0.103 (*Enu1*) for Ub. Colocalization of PAH and ubiquitin (yellow) was observed in both mutant mice, as highlighted in the inset. DAPI was used for nuclear staining (blue). Source data are provided as a Source Data file.

The reduction of total p.R261Q-PAH protein levels and increased ubiquitination observed by Western blot in *Pah-R261Q* compared to *WT* mice, indicative of instability and misfolding of this PAH variant (Fig. 3a, b), was followed-up by immunofluorescence staining of hepatic tissue which confirmed a substantial reduction in PAH protein in *Pah-R261Q* (Fig. 4). The specificity of the PAH antibody was further proven by an antigen pre-adsorption test showing almost complete loss of PAH immunoreactivity in hepatic tissue of *WT* mice after incubation with PAH antibody preabsorbed with purified recombinant PAH (Supplementary Fig. 5). Moreover, the immunofluorescence images of *Pah-R261Q* revealed scattered PAH-immunoreactivity in discrete bright points, consistent with aggregation, as well as an increase in ubiquitination signal that presented substantial colocalization with mutant PAH (Fig. 4). To investigate the mutation dependent PAH misfolding and aggregation, we also performed immunofluorescence of hepatic tissue of the *Enu1* mouse model of mild HPA, which expresses the unstable p.V106A-PAH variant, also associated with PAH instability, leading to a considerable decrease of functional PAH in the liver (5% of *WT*)^42,44,45^. *Enu1* liver also presented largely decreased and particulate PAH-immunoreactivity and increased ubiquitination. There were, however, differences in the aggregation pattern of mutant PAH between both HPA-mouse models (Fig. 4) as well as in the size of the PAH aggregates, larger in *Pah-R261Q* than in *Enu1*.

**Fig. 5:**
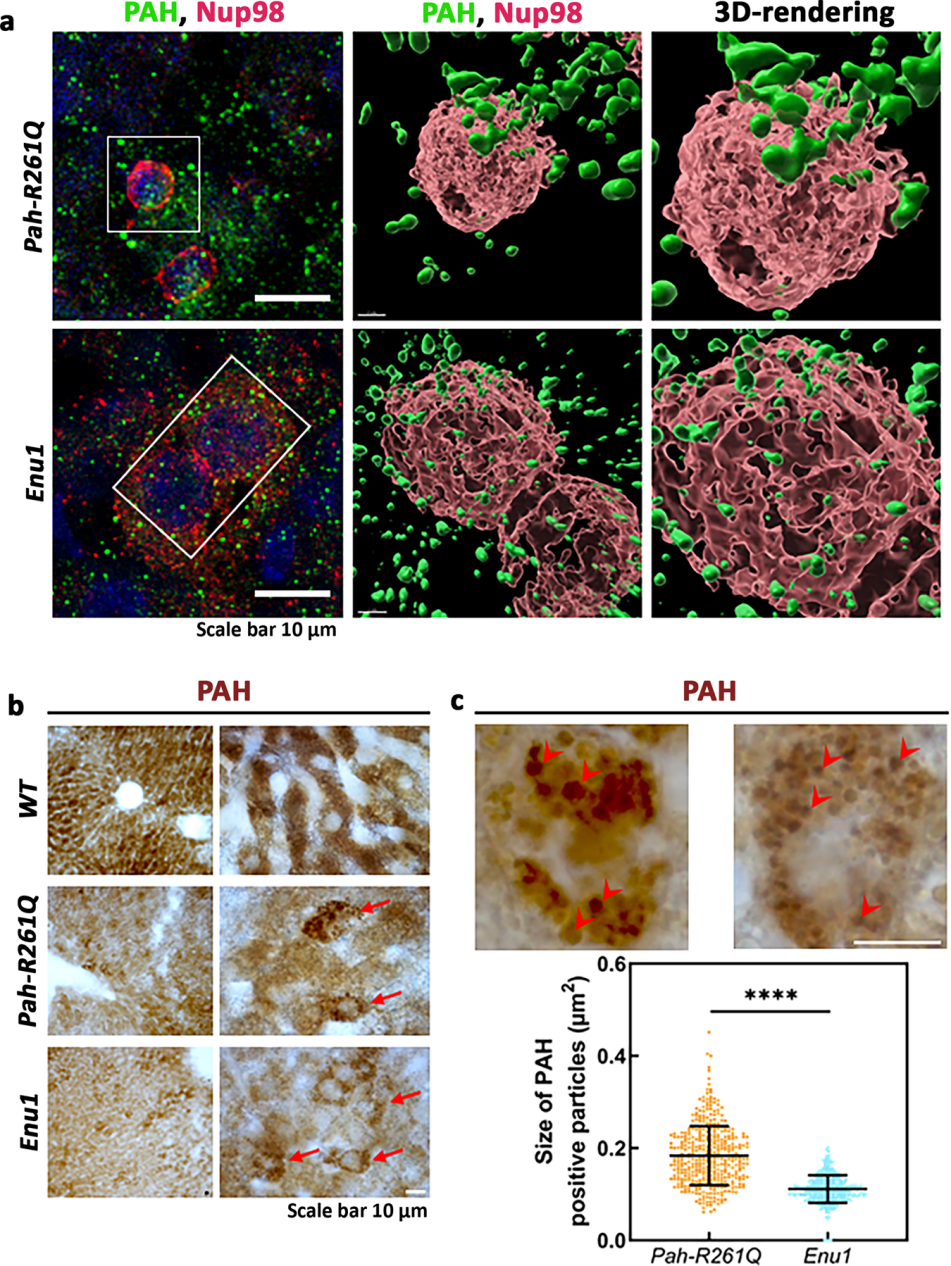
Nuclear distribution of mutant PAH protein in *Pah-R261Q* and *Enu1* mice liver. **a** Immunofluorescence of PAH (green) and the nuclear pore marker Nup98 (red) in hepatic tissue of *Enu1* and *Pah-R261Q* mice (left panels), and 3D-rendering of stacks of confocal images using the surface tool in Imaris software at two different magnifications (middle and right panels). The images reveal the subcellular distribution of PAH in the nucleus and cytoplasm of *Enu1* mice, whereas in *Pah-R261Q* mice PAH is distributed in the cytoplasm. Hoechst was used for nuclear staining (blue). **b** Immunohistochemically DAB-stained hepatocytes from *WT*, *Pah-R261Q,* and *Enu1* mice at low (left panels) and high (right panels) magnification. Arrows indicate PAH immunoreactive hepatocytes in *Pah-R261Q* and *Enu1* mice. **a**, **b** The micrographs are representative of *n* = 3 biological replicates in each mice group. **c** Two representative PAH immunoreactive hepatocytes at higher magnification in *Pah-R261Q* (left) and *Enu1* (right) mice are shown, where arrowheads point to PAH-positive particle-like structures. Measurement of PAH particle size was performed in 30 µm-thick liver sections (*n* = 10) for each mice. At least 400 single PAH-positive particles in randomly selected regions of liver sections from each mice group were analyzed, and the size distribution of PAH-positive particles in *Pah-R261Q* and *Enu1* hepatocytes is shown (lowest panel). Data are presented as mean ± SD and each dot represents a PAH-positive particle. The difference in size is statistically significant, as calculated by the two-tailed unpaired *t* test; *P* value < 0.0001 (****). Source data are provided as a Source Data file.

We further studied the distribution of PAH aggregates between the nucleus and the cytoplasm in hepatic cells. The use of the nuclear pore marker Nup98 and 3D-rendering of the stacks of confocal images revealed that the smaller aggregates of mutant PAH in *Enu1* appeared more ubiquitously distributed in hepatocytes, where they are also present in the nucleus (Fig. 5a). On the other hand, the PAH aggregates in *Pah-R261Q* did not show nuclear localization and appear distributed at the cytoplasmic side of the nuclear membrane (Fig. 5a). As fluorescence detection may alter the shape and size of macromolecules and complexes, we also performed immunohistochemistry (IHC) with optical detection by DAB staining in order to assess the size of the mutant PAH aggregates in hepatic tissue (Fig. 5b, c). The averaged area of DAB-stained particles was 0.18 ± 0.06 and 0.11 ± 0.03 (SD) µm^2^ for *Pah-R261Q* and *Enu1*, respectively (Fig. 5c).

### Amyloid-like aggregation of p.PAH-R261Q

The different size and nucleocytoplasmic distribution of the aggregates of p.R261Q and p.V106A PAH variants in *Pah-R261Q* and *Enu1* livers, respectively, suggests different misfolding and aggregation mechanisms for these two mutants. As larger aggregates, especially those with amyloid aggregation, can be cytotoxic^46^, we investigated if the mutant protein p.R261Q-PAH could aggregate through cross-β-sheet prone motives around the mutation site. Positively, in silico evaluation with the program TANGO^47^ predicted a high (>50%) propensity to form intermolecular cross-β (amyloid-like) aggregates in region 254–263 in the mutant (FLGGLAFQVF), while the same region in the WT sequence (FLGGLAFRVF) was not predicted to be prone to amyloid-like aggregation (Supplementary Fig. 1), for both the human and mouse PAH sequences. We tested the propensity of all PAH missense variants registered at BIOPKU (http://www.biopku.org), and also included p.V106A-PAH (*Enu1*), which is very rare in human^48^. The calculations supported a high susceptibility to undergo this type of aggregation for a few variants, i.e., p.E78V, p.N167Y, p.P211L, p.R261G, and p.E390G, all rare, but not for p.V106A (*Enu1*).

To confirm the formation of amyloid-like aggregates by the mutant p.R261Q-PAH we used the Amytracker^™^ 680 fluorescence assay with purified recombinant mutant protein. An accelerated formation of amyloid-like aggregates was observed for p.R261Q-PAH compared to WT-PAH (Supplementary Fig. 6a). After 5 h incubation, parallel samples of p.R261Q-PAH without Amytracker were visualized by transmission electron microscopy (TEM). Imaging revealed larger amorphous aggregates of a diameter up to >100 nm, together with small aggregates of about 20 nm diameter, whereas fibrillary structures were not observed (Supplementary Fig. 6b).

### PAH aggregates in *Pah-R261Q* mice colocalize with autophagy markers and are associated with oxidative stress

**Fig. 6:**
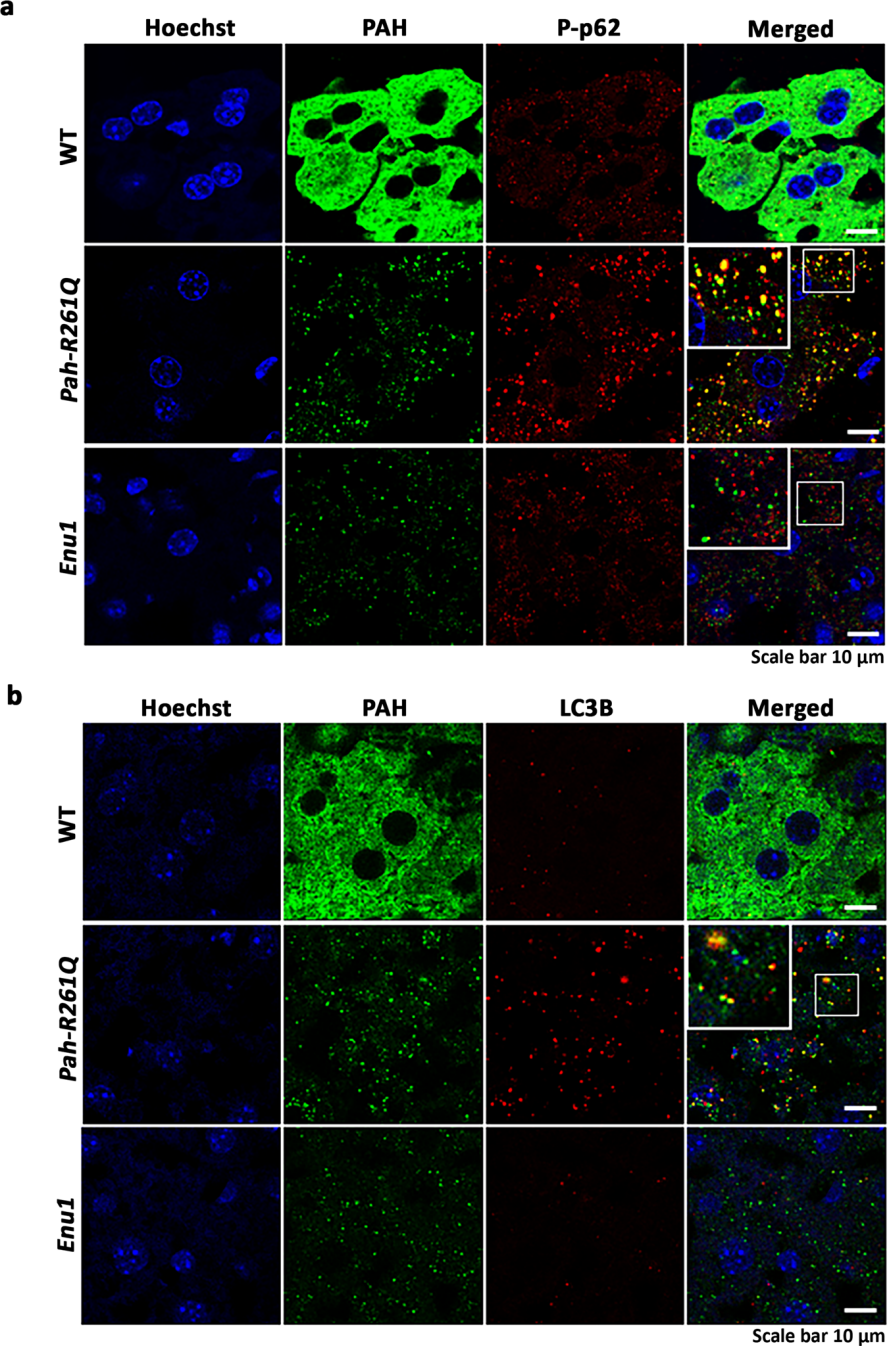
Colocalization of mutant PAH in *Pah-R261Q* mice with autophagic markers. Immunofluorescence micrographs showing the codistribution of PAH (green) with autophagy markers p62 phosphorylated at Ser403 (P-p62, red) in (**a**) or LC3 (red) in (**b**) in hepatic tissue from *WT*, *Pah-R261Q* and *Enu1* mice. Both markers were increased in *Pah-R261Q* when compared to both *WT* and *Enu1*. The fluorescence intensity (mean ± SD) calculated in 14 stacks of confocal images, relative to *WT* (=1), was 1.326 ± 0.121 (*Pah-R261Q*) and 0.778 ± 0.158 (*Enu1*) for P-p62 (**a**), and 2.277 ± 0.174 (*Pah-R261Q*) and 1.535 ± 0.175 (*Enu1*) for LC3 (**b**). *Pah-R261Q* but not *WT* or *Enu1* showed clear colocalization (yellow) of PAH with both P-p62 (**a**) and LC3 (**b**), as highlighted in the insets. Hoechst was used for nuclear staining (blue). All micrographs are representative for *n* = 3 biological replicates in each mice group. Source data are provided as a Source Data file.

Larger aggregates are commonly processed by autophagy rather than by the ubiquitin-dependent proteasome system (UPS)^49^. We thus performed immunofluorescence microscopy of liver samples of *WT*, *Pah-R261Q*, and *Enu1* mice with autophagy markers Ser403-phosphorylated p62 protein (p62/SQSTM1 (sequestosome-1)), a selective receptor and marker for autophagic clearance^50,51^. The level of phosphorylated p62 (P-p62) was indeed much higher in *Pah-R261Q* than in *Enu1* mice, which presented similar immunodetected levels as in *WT* mice (Fig. 6a). Immunofluorescence staining with the standard marker for autophagosomes LC3 was also increased in *Pah-R261Q,* but not in *Enu1*, compared with *WT* (Fig. 6b). Both autophagy markers P-p62 and LC3 presented colocalization with mutant PAH in *Pah-R261*Q mice (Fig. 6a, b). These results suggest that the larger PAH aggregates in *Pah-R261Q*, but not the smaller aggregates in *Enu1*, engage the autophagic system. Moreover, we also performed TEM imaging of hepatic tissue, which showed hepatocytes with normal cell and organelle morphology in *Pah-R261Q* and no abnormality in nuclei or the nuclear membrane (Supplementary Fig. 7a). We noticed an increased number of lysosomes and of autophagic structures, i.e., double-membrane autophagosomes and autolysosomes (Supplementary Fig. 7b) and exhaustive counting of these structures confirmed that they were increased in *Pah-R261Q* compared to *WT* mice whereas no difference in the number of peroxisomes and lipid drops was found (Supplementary Fig. 7c).

The nuclear quality control system customarily collaborates on the degradation of misfolded cytoplasmic proteins and small aggregates^52^. However, larger aggregates may hinder nuclear uptake^46^, leading to toxic accumulation of the aggregates in the cytoplasm, saturation of the autophagy system, and increased oxidative stress^49,53^. For some aggregation disorders associated with the formation of amyloid fibrils by Tau, such as polyglutamine diseases, AD and Tau-dementia, invaginations or indentations of the nuclear membrane, filled by fibrillary rods, as well as nuclear pore pathology are observed^54,55^. These alterations are also observed in ALS/FTD caused by aggregation of TDP-43 in non-fibrillary oligomeric amyloid-like aggregates^56^ and seem related with the interference of the proteins with the nucleocytoplasmic system, e.g., microtubules in the case of Tau^54^ or the nuclear transport machinery in the case of TDP-43^56^, resulting in the invaginations. The lack of nuclear invaginations in *Pah-R261Q* is in accordance with the non-fibrillary nature of the PAH aggregates and with PAH being a cytoplasmic enzyme with no known functional association with the nucleocytoplasmic system.

Despite a lack of significant disturbances in nuclear morphology in *Pah-R261Q,* it appears that the aggregates of p.R261Q-PAH do not enter the nucleus (Fig. 5a), which can be associated with overload of the cytoplasmic quality control system and oxidative stress. We measured the total equivalent antioxidant capacity (TEAC) in liver lysates of both HPA models (*Enu1* and *Pah-R261Q*) and *WT* mice by the Trolox assay. *Pah-R261Q* but not *Enu1* presented elevated TEAC values compared with *WT* (Supplementary Fig. 8), indicating a specific upregulation of the antioxidant response in *Pah-R261Q* mice in agreement with the metabolic changes associated with oxidative stress (see above and Table 1).

### Gene (mRNA) expression assays

Finally, ten genes related to the PAH system, protein quality control, and oxidative stress pathways were selected and subjected to analysis by quantitative TaqMan mRNA expression in liver extracts (RT-qPCR; results overview in Table 2). In absolute terms, *Pah* and *Hsc70* showed the highest levels of gene expression. Comparatively, for *Pah-R261Q* vs. *WT* mice, we found the following: (i) *Pah* expression was not altered; (ii) expression of the *GCH1-feedback regulatory* (*Gchfr*) gene was upregulated; (iii) the PAH specific Hsp40 co-chaperone *Dnajc12* exhibited increased expression levels while two heat shock family members, namely the transcription factor *Hsf1* and the molecular chaperone *Hsp70* were downregulated, and the expression of the constitutive *Hsc70* was unmodified; (iv) no significant changes in expression levels were identified for targets associated with protein degradation, *Stub1* (coding for the co-chaperone CHIP, with ubiquitin ligase activity) and the autophagy marker *Sqstm1* (coding for p62/SQSTM1), and for the oxidative stress-responsive transcription factor *Ap-1*. The implication of these results together with the other findings in this work are discussed below.

**Table 2:** Relative mRNA quantification for selected genes in liver of *WT* and *Pah-R261Q* mice.

Gene (name)	*Pah* genotype	Expression level (relative to *WT* mice, defined as 1)	Fold change^a^	*p* Value^b^
*Pah*	*WT*	1.0000 (0.7878 ± 1.2693)	1.15	0.2273
	*R261Q*	1.1524 (1.0967 ± 1.2110)		
*Gch1*	*WT*	0.2512 (0.2146 ± 0.2939)	1.08	0.5440
	*R261Q*	0.2714 (0.2115 ± 0.3483)		
***Gchfr***	***WT***	**0.0163 (0.0131** ± **0.0203)**	**1.70**	**0.0024**
	***R261Q***	**0.0277 (0.0228** ± **0.0336)**		
***Dnajc12***	***WT***	**0.0040 (0.0030** ± **0.0053)**	**1.78**	**0.0048**
	***R261Q***	**0.0071 (0.0057** ± **0.0088)**		
***Hsp70***	***WT***	**0.0010 (0.0007** ± **0.0015)**	**0.31**	**0.0012**
	***R261Q***	**0.0003 (0.0002** ± **0.0005)**		
*Hsc70*	*WT*	0.9704 (0.7478 ± 1.2592)	1.34	0.0855
	*R261Q*	1.2983 (1.0279 ± 1.6399)		
***Hsf1***	***WT***	**0.0280 (0.0251** ± **0.0313)**	**0.65**	**0.0054**
	***R261Q***	**0.0183 (0.0140** ± **0.0238)**		
*Stub1*	*WT*	0.0156 (0.0126 ± 0.0193)	1.22	0.0808
	*R261Q*	0.0191 (0.0175 ± 0.0208)		
*Sqstm1*	*WT*	0.2483 (0.2203 ± 0.2799)	1.04	0.6389
	*R261Q*	0.2571 (0.2287 ± 0.2892)		
*Ap-1*	*WT*	0.0163 (0.0140 ± 0.0189)	1.0	0.9815
	*R261Q*	0.0163 (0.0149 ± 0.0179)		

^a^Fold change in *Pah-R261Q* mice relative to *WT* mice.

^b^Two-tailed *p* values for differences between both mice groups, obtained from the Mann Whitney *U* test.

Genes upregulated or downregulated in *Pah-R261Q* (*n* = 5 mice) compared to *WT* (*n* = 6 mice) (*p* < 0.05) are highlighted in bold text.

## Discussion

Mouse models of genetic diseases do not always entirely recapitulate the main phenotypic characteristics found in patients^9^. The *Pah*-*R261Q* knock-in mouse that carries a frequent mutation in HPA/PKU patients exhibits reduced total hepatic PAH activity and presents phenotypic traits characteristic of homozygous patients with the *R261Q:R261Q* genotype, such as increased L-Phe and decreased L-Trp and L-Tyr in blood compared to *WT*, sensitivity to L-Phe challenge, and responsiveness to BH_4_ supplementation (http://www.biopku.org)^8,16^. Moreover, there is similar PAH residual activity (~15% of *WT*) in both *Pah-R261Q* mice and homozygous humans^16^, however, we encountered a remarkable difference between absolute blood L-Phe-levels in mice and patients. While patients present metabolic phenotypes from mild PKU to classic PKU (off-diet blood L-Phe values > 600 µmol/L) (247 records in http://www.biopku.org; see also refs. ^16,17^), our mouse model exhibited very mild HPA (~110 µmol L-Phe/L). Thus, for the same *PAH* genotype with similar remaining activity, the blood L-Phe concentration (metabolic phenotype) is higher in humans, which might be explained by a lower steady-state level of hepatic PAH in humans compared with mice. There is a high similarity in sequence, structure, specific activity and regulatory properties of human and mouse PAH, and thus different PAH amounts between rodents and humans have been associated with differences in the rate of transcription, translation, and/or protein homeostasis^57^. Nevertheless, based on the similar propensity to aggregate through a cross-β-sheet formation for human and mouse PAH around the mutation area (Supplementary Fig. 1), it is very probable that similar amyloid-like soluble aggregates are formed in the liver of patients with the Arg261 → Gln mutation.

The *Pah-R261Q* mouse appears to offer considerable potential for mechanistic and therapeutic investigations as it presents with a tunable blood L-Phe concentration. By applying an L-Phe challenge, we could transiently attain L-Phe concentrations characteristic of PKU, and by adjusting the L-Phe concentration and length of supplementation, it might be possible to modulate the metabolic phenotype and fully develop the capacity of this mouse model as a prototype to study a range of mild to severe forms of HPA. Furthermore, upon L-Phe challenge, the resulting transient HPA in *Pah-R261Q* is responsive to treatment with BH_4_ (Kuvan®). Consequently, this mouse model can contribute to evaluate protocols and understand the multifactorial mechanisms for BH_4_ responsiveness in mice, including the increase of total PAH enzyme activity  by PAH variant stabilization through protection against oxidative damage and proteolytic degradation, thus prolonging the half-life of PAH as seen in vitro^19^.

The large variability and spectrum of metabolic phenotypes presented by homozygous *PAH-R261Q* patients^15–17^ are thus far not observed in the mouse model. *Pah-R261Q* mice, in fact, present low variation in the basal concentration of blood L-Phe and other parameters measured in this work. The proteostasis network that maintains the synthesis, folding, localization, and degradation of proteins, and counteracts the effect of aggregates, involves a large number of protein components that are regulated at the cellular, tissue, and organismal level^53^. This complex proteostasis network provides additional polymorphic modifier variants that contribute to the broader phenotypic spectrum in patients with unstable PAH variants, but not necessarily in the mouse model where a uniform genetic background has been achieved by careful backcrossing.

In PKU, the neurological defects include monoamine neurotransmitter deficiencies, which are fully manifested in the classical PKU (*Enu2*) mouse that is almost devoid of PAH activity^26^. The *Enu1/2* mouse, which presents 2.5% of regular PAH activity and blood L-Phe levels just slightly higher (~150 µmol/L) than in *Pah-R261Q*, also shows a decrease in brain serotonin and 5HIAA^58^. However, the remaining 16% PAH activity in *Pah-R261Q* mice appears high enough to result in apparently normal L-Phe catabolism and monoamine neurotransmitter synthesis, as well as in absence of detectable neurological deficiencies. Nevertheless, despite their mild HPA, *Pah-R261Q* mice manifested several biomarkers and indicators of adiposity and altered lipid metabolism, and oxidative stress. These traits have been previously observed in patients and animal models of PKU where they have been related to neurotoxic HPA levels and (micro)nutritional deficiencies of the L-Phe-free diet^59,60^, factors that are absent in this case. Further evidence suggests that the increased weight in *Pah-R261Q* males is not a direct consequence of elevated L-Phe levels; the classical PKU model *Enu2* is underweight, despite its tenfold higher blood L-Phe levels than in *Pah-R261Q*^26^. The molecular basis behind the observed mild overweight and oxidative stress in *Pah-R261Q* mice appears to be related to a toxic aggregation of mutant PAH and contributes to the identification of a gain-of-function contribution to the HPA/PKU pathology.

The misfolding defect of the p.R261Q-PAH protein variant is manifested both in biochemical characterizations as a reduced conformational stability and accelerated degradation^18,19,23^ and in computational predictions by FoldX^15^. The Arg to Gln residue change is expected to disrupt the interdimer interactions in p.R261Q-PAH (Supplementary Fig. 1), and the area around the mutation would then become available for unspecific intersubunit interactions. Among the few HPA/PKU-associated PAH variants with a high predicted propensity for β-cross amyloid-like aggregation by in silico TANGO calculations^47^, p.R261Q-PAH is the variant with the highest allele frequency among patients.

The *Pah-R261Q* mice presented a reduction in steady-state hepatic PAH levels and PAH-specific activity, as well as increased ubiquitination of the protein in the liver. The current understanding of the loss-of-function of misfolding PAH variants is an accelerated degradation carried out preferentially by the ubiquitination-dependent proteasome system (UPS), as recently proven for a large number of PAH variants in cellular studies^61^. Our results with *Pah-R261Q* show that selective autophagy may be involved in the degradation of a PAH variant, as strongly indicated by the colocalization of markers of autophagy Ser403-phosphorylated p62 and LC3 with mutant PAH. In the case of the lightly aggregating *Enu1* variant p.V106A-PAH there is no colocalization of these markers with PAH, and the aggregates seem to enter the nucleus where they may also be degraded by the nuclear UPS. There is an intricate cross-talk between the UPS and autophagy^62,63^, and it is thus likely that PAH amyloid-like aggregates in *Pah-R261Q* that are not effectively processed by the UPS can be co-aggregated with phosphorylated p62 for autophagic processing^51,64^.

Although insoluble deposits may protect from oxidative stress^65^, amyloid-like aggregation-prone conformers—e.g., resulting from mutations—perturb cellular homeostasis and induce oxidative stress, increasing the production of reactive oxygen species (ROS) at the cellular and tissue levels^53^. Toxic aggregation in the cytoplasm overwhelms the protein quality control system, resulting in increased ROS, further exacerbation of protein aggregation^53^, and activation of p62/SQTM1 by phosphorylation^51^. Together, our results point to an oxidative and cellular stress condition in *Pah-R261Q* mice associated with a toxic aggregation of the PAH variant. The reduction of BH_4_ levels manifested in the liver of *Pah-R261Q* has also been observed in other disorders associated with oxidative stress^2^. In hepatocytes, BH_4_ also acts as the cofactor of alkylglycerol monooxygenase^2^, an enzyme involved in the degradation of ether lipids. Alteration of BH_4_ synthesis mainly affects the entire cellular lipidome^66^, providing a link between oxidative stress and alterations of lipid metabolism, the two main comorbidities postulated from the metabolic characterization of *Pah-R261Q* mice (Table 1). As part of the physiological and metabolic characterization of *Pah-R261Q,* we noted three related findings: slightly increased body weight of males (26.8 ± 0.4 vs. 25.1 ± 0.3 g (*WT*)), lower RER in the resting period, and higher serum levels of some metabolites that have been associated with adiposity and altered lipid metabolism, namely trimethyllysine, leucine, and isoleucine^31^. Although no gender-associated changes were found for any parameter or metabolite measured in this work for *Pah-R261Q* compared to *WT*, including RER and relevant metabolites, a priori indicates a similar propensity for altered metabolism in both genders, only males were heavier than their *WT* counterparts. A male-specific body weight increase due to altered lipid metabolism and adiposity has been detected in other mice and human studies, which has been associated with a different genetic architecture and potential sex chromosome effects on metabolism (reviewed in ref. ^67^).

A recent study has also reported the reduction of soluble BH_4_ in the liver lysates of *Enu1* and *Enu1/2* mice and the decrease has been linked to the entrapment of the cofactor in aggregates of the p.V106A-PAH variant^45^, resulting in a secondary BH_4_-deficiency. Here, we measured total BH_4_ in non-centrifuged homogenates, showing a net reduction in *Pah-R261Q* mice, which is also supported by the upregulation of *Gchfr-*mRNA (Table 2). Furthermore, no decrease of hepatic BH_4_ was measured by the same method in liver *Enu1/2* mice^43^, supporting that the BH_4_ reduction in *Pah-R261Q* is PAH mutation-specific and associated with the formation of large amyloid-like aggregates and oxidative stress.

Oxidative stress in *Pah-R261Q* mice elicits the activation of the antioxidant response, as seen by increased serum levels of β-hydroxybutyrate^33^, as well as an increase in total antioxidant capacity measured by the Trolox assay. Likewise, the reduction of quinolinic acid could also be linked to an increased synthesis of NAD^+^, which polymerizes to poly ADP-ribose for the protection of DNA in case of oxidative stress^36,37^. Conversely, increased β-oxidation (demonstrated by lower RER and elevated β-hydroxybutyrate) and the concomitant increase in anaplerosis, supported by the observed reduction in α-ketoglutaric acid and other anaplerotic amino acids and metabolites (Table 1) has also been shown to cause an increase in oxidative stress and inflammation^38^. Another sign of oxidative stress in *Pah-R261Q* is the upregulation of *Dnajc12*, the specific co-chaperone of the aromatic amino acid hydroxylases, which was not altered in *Enu1*^42^. Interestingly, overexpression of *Dnajc12* has been associated with oxidative stress^68,69^. On the other hand, the downregulation of both the transcription factor *Hsf1* and the chaperone *Hsp70* appears somehow counterintuitive in this context since *Hsf1* in mammals is the primary regulator of the heat shock response, which is activated by cellular stress and elicits transcriptional upregulation of major HSPs, notably *Hsp70*^70^. In contrast, downregulation of *Hsf1* and consequent reduction in expression of HSF1 target genes is observed in neurodegenerative diseases such as Parkinson’s, Alzheimer’s, and Huntington’s diseases, characterized by toxic amyloid deposits^71^, and a similar downregulation of *Hsf1* and *Hsp70* is also observed in this case.

In conclusion, the observation of amyloid-like PAH aggregates in the liver of the *Pah-R261Q* mouse introduces the concept of toxic gain-of-function for specific PKU-associated mutations. Overall, our results suggest that the lipid profile alterations and oxidative stress found in these mice are linked to intracellular toxic aggregation of the p.R261Q-PAH variant rather than to the severity of the HPA and/or the diet. The *Pah-R261Q* mouse model thus represents a unique research tool to support the evaluation and discovery of additional biomarkers in PKU and to investigate mutation-specific comorbidities, of benefit to the large number of PKU patients harboring the *R261Q* mutation. Interestingly, recent studies aiming to assess the prevalence of comorbid associations among large groups of adult PKU patients are starting to reveal several conditions in addition to the known neuropsychiatric disorders, including overweight and renal and cardiovascular dysfunctions^5,6,72^. A proper patient stratification that takes into account the predisposition of the coded PAH variants to amyloid-like aggregation is expected to result in a better association of the comorbidities and improved patient-tailored treatment, encouraging follow-up investigations of the PAH aggregates. Lastly, this mouse model might contribute to investigations on pharmacological chaperone-based therapies targeting unstable PAH variants.

## Methods

### Materials

All chemicals in this section were acquired from Sigma-Aldrich unless otherwise indicated. Animals evenly matched for sex were distributed in each group for the different experiments presented. Recombinant human WT-PAH and p.R261Q-PAH proteins were expressed and purified using the pMAL expression vectors^18,19^ coding for the fusion protein maltose-binding protein (MBP)-(pep)xa-PAH), where (pep)xa is the cleavage site for the restriction protease Factor Xa. The fusion proteins were expressed in *Escherichia coli* TB1 cells at 28 °C for 16–18 h with 1 mM IPTG and purified by affinity chromatography with amylose resin (New England Biolabs) with elution with 10 mM maltose. The fusion proteins were cleaved for 3 h with Factor Xa (New England Biolabs) at a protease:PAH ratio of 1:300. The tetrameric purified WT-PAH and p.R261Q-PAH proteins were isolated by size exclusion chromatography on a Superdex HiLoad 16/600 200 column (GE Healthcare) in 20 mM Hepes pH 7, 200 mM NaCl.

### Generation of *Pah-R261Q* knock-in mouse

The constitutive knock-in mouse model was generated by Taconic Biosciences GmbH (Köln, Germany) via CRISPR/Cas9-mediated gene editing. The guide RNA target sequence + protospacer adjacent motif (PAM) sequence 5′-AGTGGAAG_ACTCGGAAGGCC_AGG-3′ (non-seed sequence_seed sequence_PAM) was designed and guide RNA was prepared as a hybrid of CRISPR-RNA (crRNA; 6 ng/µl) (Dharmacon, Lafayette, USA) and trans-activating crRNA (tracrRNA; 10.5 ng/µl) (Dharmacon, Lafayette, USA). The guide RNA was co-injected into C57BL/6N zygotes - essentially as described^20^—along with Cas9 protein (55 ng/µl; New England Biolabs, Ipswich, USA) and homology-directed repair (HDR) oligonucleotide (5′-GCTTAGATCCATGCCTAATGTACTGTGTGCAGTGGAAGACTTGGAATGCCAGGCCACCCAAGAAATCTCGAGACGACAGTAAGCCAG-3′) (100 ng/µl), from Integrated DNA Technologies, Coralville, USA. This HDR oligonucleotide harbored the point mutation c.782 G > A (p.Arg261Gln) to be introduced in exon 7 of the *Pah* gene on murine chromosome 10 (point mutation and exon annotation according to NCBI transcript NM_008777.3) as well as a silent mutation (c.777C > A) to create an additional restriction site (*Bsm*I) for analytical purposes (Supplementary Fig. 2). These CRISPR reagents were microinjected (until the pronucleus swells up, typically ~1 pl) into 304 mouse embryos resulting in a total of 51 pups born, and three animals (5.9%) displayed positive detection of the *R261Q* knock-in allele.

Confirmation of the on-target mutation was completed as detailed^73,74^ whereas verification of the absence of undesired potential off-target modifications was achieved by heteroduplex mobility assays conducted on the top 16 hits originated from BLAST analysis of the guide RNA onto *M. musculus* genome assembly (GRCm38/mm10) (Supplementary Table 1).

### Animal husbandry and colony expansion

The animal studies were approved by the Norwegian Food Safety Authority and performed at the Laboratory Animal Facility, University of Bergen, according to the guidelines and standards from the Regulation on the use of animals in the research of this institution. Mice were housed in a temperature-controlled (21 °C and 50% air humidity) environment with 12 h light/dark cycles. Food (standard chow pellets) and water were available ad libitum.

The colony was continuously backcrossed to avoid genomic drift. Every 6 months wild-type mice (C57BL/6J, males and females every second time) were bought from an approved vendor (Charles River) and bred with heterozygous *Pah*^*R261Q/WT*^ mice. Six to eight weeks old heterozygous *Pah*^*R261Q/WT*^ siblings from this backcrossing and breeding were used to produce homozygous *Pah*^*R261Q/R261Q*^ mice. The resultant *Pah*^*R261Q/WT*^ and *Pah*^*WT/WT*^ mice (siblings) from the breeding were utilized as control counterparts. This method of breeding is recommended to maintain the strain genetic integrity. We make sure to avoid going beyond three generations of inbreeding (F3) before we reset the generation by using new breeding animals from the backcrossing. The strain has been backcrossed eight times (N8) and the latest mice that have been used in the experiments presented in this work are from generation N8F3.

### Genotyping

In order to determine the mouse genotype, ear biopsies were collected and DNA was extracted and purified from these tissue samples using the DNeasy Blood and Tissue kit (QIAGEN) following the manufacturer’s instructions. After that, DNA was amplified by standard PCR (See primers in Table S4), initial denaturation at 95 °C/5 min followed by 35 cycles of denaturation-annealing-extension, 95 °C/30 s, 60 °C/30 s and 72 °C/1 min, with a final extension at 72 °C for 10 min) using Taq Polymerase (New England BioLabs). The PCR product was then incubated with the endonuclease *Bsm*I (New England BioLabs) for 15 min at 65 °C. Finally, the digestion fragments were resolved in 2.5% agarose gel electrophoresis (1× TAE buffer/90 min/90 v) and bands visualized in a ChemiDoc XRS (Bio-Rad Laboratories) imaging system.

### Metabolic cage

The physiological parameters: rate of O_2_ and CO_2_ consumption, food intake and activity of *Pah-R261Q* and control *WT* mice (4–5-month old; *n* = 3–5 mice per group, 121 observations/animal) were directly determined using the Oxymax-Comprehensive lab animal system (CLAMS, Columbus Instruments), with data being recorded for 36 h after an acclimatization period of 12 h. Other valuable calorimetric properties such as respiratory exchange rate (RER) and energy expenditure were indirectly calculated using Lusk classical equations provided in the Oxymax processing software.

### Rotarod performance test

The assessment of motor function was conducted on a rotarod instrument (Harvard Apparatus) consisting of a 5 cm plastic grooved rod and a platform situated approximately 25 cm below equipped with a lever to trigger the recording for the time of fall. *Pah-R261Q* and control *WT* mice (3 months old; *n* = 10–11 mice per group) were, initially, habituated to the setting and trained to stay on the rod for 1 min at a constant speed of 5 rpm. Trained mice were then placed again on the rod with a gradual acceleration from an initial 4 rpm to a final 40 rpm speed over a 5 min testing period. The latency of fall as well as rod revolutions at fall was logged, and trials were repeated three times, separated by a 15 min break.

### L-Phe challenge and BH_4_-responsiveness

Adult mice (3–4-month-old) were employed in all the animal experiments of this section. For the effect of L-Phe challenge, intraperitoneal injection of an L-Phe solution (200 µg/g body weight) to *Pah*^*R261Q/R261Q*^, *Pah*^*R261Q/WT*^, and *Pah*^*WT/WT*^ mice (*n* = 23, 6 and 4 mice per group, respectively) was followed by whole blood sampling from the saphenous vein at time points: 1 day before injection (baseline level) and 35, 85, 150, and 300 min after injection. For BH_4_-responsiveness, a treatment solution of BH_4_ (Schircks Laboratories) (12.73 mM BH_4_, 2% ascorbic acid, and 10% DMSO; in a dosage of 20 mg BH_4_/kg body weight) or placebo solution (2% ascorbic acid and 10% DMSO), were injected into *Pah-R261Q* mice (*n* = 5–6 mice per group, per experiment) intraperitoneally twice a day^41^ for 4 days, before conducting the L-Phe challenge protocol as indicated above. The obtained blood samples were collected onto filter paper cards (PerkinElmer) according to the vendor’s guidelines wherein L-Phe was measured by tandem mass spectrometry in, at minimum, 3 h fasted mice.

### Metabolic markers analysis

In order to obtain serum specimens, whole blood was collected by cardiac puncture and 1 mL was transferred to a microcentrifuge tube. Subsequently, blood samples were left for 45 min at room temperature to facilitate coagulation, preceding two consecutive centrifugation steps (2.100 rcf, 10 min, and 4 °C) where the respective supernatants were pipetted out to a clean tube. The isolated serum fractions were stored at −80 °C until examination. The extracted serum samples from *Pah-R261Q* and *WT* mice (4–5 months old; *n* = 19 mice per group) were subjected to an exhaustive analysis to determine the concentration of 72 relevant metabolic biomarkers. Analyses were performed at Bevital (http://bevital.no/) across four different platforms supporting high-throughput multi-analyte assays. A combination of chromatographic techniques with mass spectrometry detection (GC–MS/MS and LC–MS/MS) was applied (See references for individual experimental protocols in Supplementary Table 2).

### Preparation of liver and brain lysates

Mice (age and numbers indicated in the specific studies) were sacrificed in a carbon dioxide euthanize chamber. Immediately after, the brain and liver were surgically excised and snap-frozen in liquid nitrogen, and tissue was manually ground into a fine powder and stored in aliquots at −80 °C. Liver homogenates from the ground aliquots (~200 mg powder) were prepared by adding 800 µL of a lysis buffer solution containing 1× PBS and protease inhibitor cocktail (Roche), a 5 mm diameter stainless steel bead (Qiagen), and a mechanical disruption step in a Tissue Lyser II (Qiagen) instrument (2× 1 min 30 s, 20 Hz). Cellular debris was then removed through centrifugation (20.000 rcf, 20 min) to obtain a clear supernatant. The total protein concentration of the liver lysates was determined in a Direct Detect infrared spectrometer (Millipore). For the Trolox equivalent antioxidant capacity assay, 600 µL of identical lysis buffer, a mechanical homogenization with a pellet pestle sitting on ice, and a modified centrifugation step (18.000 rcf, 10 min) was preferred.

Brain extracts were prepared by powder homogenization in 10× volume of 50 mM Tris-HCl, pH 7.5, 100 mM KCl, 1 mM EDTA, 1 mM DTT, 1 μM leupeptin, 1 μM pepstatin, and 200 μM PMSF, at otherwise identical experimental conditions as standard liver extracts (see above).

### Neurotransmitters, BH_4_, and amino acid determination in tissues

The relative levels of BH_4_ in liver and brain and of monoamine neurotransmitter metabolites in the brain of *Pah-R261Q* and *WT* (*n* = 5–6 mice per group) were measured in liver and brain lysates of 3–4-month-old mice as reported^58^. Briefly, tissue lysates were oxidized for 60 min in the dark by 0.5 g/L iodine and 0.1 g/L potassium iodide in 0.1 M HCl. The oxidation was stopped by adding 2 g/L ascorbic acid and adjusting the pH to 8.5 with NaOH before incubation with calf intestine alkaline phosphatase (Roche Applied Science) at 37 °C for 1 h. The lysates were then adjusted to pH 2 with HCl and deproteinized (Ultrafree-MC filters, Millipore) before BH_4_ and neurotransmitters were measured by HPLC^75^.

The relative levels of the amino acids L-Phe, L-Tyr, and L-Trp of *Pah-R261Q* and *WT* (*n* = 5–6 mice per group) were measured in liver and brain lysates of 3–4-month-old mice as reported^76^. Samples were prepared according to the Phenomenex EZ:faast^™^ kit’s manual, with the following modifications: prior to amino acid extraction and derivatization, 20 μL of each internal standard solution containing 100 μmol/L Phe-d5 and 20 μmol/L Tyr-d4 (in 50 mmol/L HCl) were added to 20 μL of sample lysate. Using the kit’s reagents, the amino acids were derivatized with propyl chloroformate resulting in the addition of propyl formate at the amine moiety and a propyl group at the carboxylic end of the amino acids, respectively. The hydroxy group of Tyr was also derivatized by the addition of a propyl formate group, and the amino acids were then measured by LC–ESI–MSMS^76^.

### PAH enzymatic activity assay

Liver lysates were first loaded into 0.5 mL Zeba-Spin desalting columns (7.000 Da cutoff; ThermoFisher Scientific), previously equilibrated with 20 mM HEPES, pH 7.0, 200 mM NaCl, and protease inhibitor cocktail solution, and centrifuged (1.700 rcf) for 2 min. PAH activity in the homogenates was measured at 25 °C using 5–20 µg of total protein in each assay, with 1 mM L‐Phe in 20 mM Na–Hepes, 0.2 M NaCl, pH 7.0, containing catalase (0.04 mg/ml). After 4 min preincubation at 25 °C, ferrous ammonium sulfate (100 µM) was added, and the reaction triggered after 1 min by adding 200 µM BH_4_ and 5 mM DTT (final concentrations in the assay). The reactions were allowed to run for 2 min and stopped with 2% acetic acid in ethanol. Under these conditions, PAH activity was linear to the amount of protein in the extracts. L‐Tyr formed was quantified by HPLC with fluorimetric detection.

### Immunoblotting

Protein immunodetection was performed by Western blot. Total protein (2.5 µg/well) was separated using 10% polyacrylamide gel and immunodetected by using primary antibodies, 1:5000 for primary antibody α-PAH (1:5000; Millipore-MAB5278), α-ubiquitin (1:500; Thermo Fisher Scientific-131600); α-glyceraldehyde 3-phosphate dehydrogenase (GAPDH) (1:1000; Abcam-ab9485), and 1:2500 for secondary antibodies goat anti-mouse (GAM) (Bio-Rad Laboratories) and goat anti-rabbit (GAR) (Bio-Rad Laboratories), conjugated to horseradish peroxidase. Quantification of non-ubiquitinated and mono-ubiquitinated  PAH and GAPDH proteins was performed by gel band densitometry.

### Immunofluorescence and IHC

Mice (4–5-month-old) were anesthetized with pentobarbital (20 mg/kg, IP), and transcardially perfused with 50 ml of warm saline (0.9%, 37 °C), followed by 50 ml of warm paraformaldehyde (4%, 37 °C) in 0.16 M phosphate buffer (PBS; pH 7.2). The hepatic tissue was dissected out and postfixed in the same fixative for 3 h at 4 °C and subsequently stored in 20% sucrose diluted with PBS containing 0.01% sodium azide (Sigma) and 0.02% Bacitracin (Sigma) at 4 °C overnight. Tissues were embedded with Optimal cutting temperature (OCT) compound (Tissue Tek, Miles Laboratories, Elkhart, Ind., USA), frozen and cut in a cryostat (Microm, Heidelberg, Germany) at 20 or 50 μm thickness, collected and stored free-floating in PBS at 4 °C or mounted onto SuperFrost Plus microscope slides (ThermoFisher Scientific), dried at room temperature for 30 min and stored at −80 °C until use.

For immunofluorescence, Triton-X 100 (0.5%) was used to permeabilize the tissues followed by blocking with 5% FBS in PBS for 1 h prior to the primary antibody incubation. Tissues were then incubated overnight at 4 °C in a humidity chamber with the correponding primary antibody, α-PAH (1:100; Abcam-ab178430), α-Ubiquitin N-terminal (1:200; ABIN350072), anti-NUP98 (1:100; Abcam-ab50610), α-phospho-p62 (S403) (1:200; MBL-D343-3), and goat anti-LC3B (1:100; Signalway antibody-C48312), in 10% (w/v) NGS, 1× PBS, pH 7.4 solution. Then, a 30 min incubation in a humidity chamber at 37 °C was carried out with the corresponding secondary antibody, donkey anti-rabbit IgG H&L (1:200; Alexafluor 488, Invitrogen-A21206), donkey anti-goat IgG H&L (1:400; Alexafluor 555, Abcam-ab150130), donkey anti-goat IgG (1:200; Cy3 conjugate, Millipore-AP180C), goat anti-rat IgG H&L (1:100; TRITC, Jackson immunoresearch-112-025-143), goat anti-rabbit IgG H&L (1:200; Alexafluor 647, Invitrogen-A21245), donkey anti-rat IgG H&L (1:200; Alexafluor 488, ThermoFisher Scientific-A21208), in the same blocking solution. A washing step of 15 min with 1× PBS, pH 7.4 was included prior to and after each incubation period. Hoechst or DAPI, as indicated, were used to counterstain the nucleus for 30 min at RT followed by washing in PBS and mounted with DABCO mounting media. Images were acquired by using Leica TCS SP5 confocal microscope (Leica Microsystem GmbH) using a pinhole airy 1 and a 63×, 1.4 numeric aperture oil immersion objective. Acquired images were processed using the LAS AF Lite software (Leica Microsystem). For each sample, a stack of images (*n* = 14) with a step-size of either 290 or 170 nm was taken. Fluorescence intensity measurements were performed using Fiji freeware using the stacks of confocal images. Integrated density values of each stack were used to compare the relative fluorescence intensity of the samples. 3D rendering of the confocal images (z-stacks) was performed by using image analysis software Imaris (Bitplane Inc.). The nuclei marked with Nup98 (red) and PAH (green) were reconstructed by using the surface tool.

For immunohistochemistry (IHC), the stored free-floating sections were employed. Sections were first rinsed in 0.3% H_2_O_2_ in PBS for 10 min at RT for quenching endogenous peroxidase activity, and then incubated with blocking buffer containing Blocking Serum (VECTOR Laboratories), 0.5% Triton X-100 (Sigma), and 5% bovine serum albumin (Sigma-Aldrich) in PBS for 1 h at RT. Sections were incubated for 45 min with primary antibody α-PAH (1:800, Abcam- ab191415) and 30 min with goat anti-rabbit IgG H + L (1:200, HRP, BioRad-1706515) secondary antibody, and followed by a 30-min incubation with prepared VECTASTAIN Elite ABC (VECTOR Laboratories). The sections were immersed in a peroxidase substrate solution (DAB, Sigma) for 7–8 min and washed with water, mounted on Super Frost slides, and coverslipped with glycerol/PBS (9:1) containing 0.1% para-phenylenediamine. Finally, the sections were analyzed and images were captured using a Leica microscope equipped with a Leica camera. The average size and size distribution of PAH-positive “particles” in the cytoplasm of hepatic cells was quantified using ImageJ software. As the PAH staining pattern in the light microscope (objective 100×) was evenly distributed in *WT* mouse liver, the quantification was performed on liver sections of *Pah-R261Q* and *Enu1*. Ten liver sections (30 µm thickness) were randomly selected from each animal. PAH-positive particles were analyzed in randomly selected regions of the sections. Only single PAH-positive particles were analyzed and at least 400 particles from each group, from at least 10–12 hepatic cells, were counted.

### Amyloid-like aggregation assay (Amytracker)

Amyloid detection was carried out by recording the fluorescence emission of amyloid ligand heptamer formyl thiophene acetic acid (Amytracker™ 680; Ebba Biotech) at 680 nm in a 96-well plate, black F-bottom (Griener Bio-one), for 24 h at 37 °C in a multimode microplate reader (Tecan spark), with excitation at 540 nm. The time course of the fluorescence intensity for purified WT-PAH and p.R261Q-PAH (20 µM subunit) with Amytracker^™^ 680 (1:1000) was acquired in 20 mM HEPES, 150 mM NaCl, pH 6.0. Samples lacking protein were used as controls and normalized. Three measurements were carried out for each protein.

### Transmission electron microscopy (TEM)

For TEM of hepatic tissue, 5-month-old mice (*WT* and *Pah-R261Q*) were anesthetized with isoflurane and transcardially perfused with 50 ml of warm saline (0.9%, 37 °C), followed by 50 ml of 2.5% glutaraldehyde (diluted in a 0,1 M sodium cacodylate buffer) at RT. The hepatic tissue was dissected out and postfixed in the same fixative for 24 h at 4 °C. The tissues were then transferred to 0.1 M sodium cacodylate buffer and kept at 4 °C. Postfixation was performed for 1 h (on ice) in 1% osmium tetroxide (EMS # 19134) diluted in 0.1 M sodium cacodylate buffer, followed by two washing steps. The samples were then dehydrated using a graded ethanol series (30%, 50%, 70%, 96%, and 3 × 100%) before transferred to a 1:1 solution of 100% ethanol:propylene oxide (15 min). Samples were then transferred to 100% propylene oxide (15 min) before gradually introducing agar 100 resin (AgarScientific R1031) drop by drop over the next hours. Samples were then transferred to a small drop of 100% resin and excess propylene oxide was allowed to evaporate (1 h). Samples were then transferred to 100% resin and placed in molds and left at RT overnight. The molds were then placed at 60 °C for 48 h to polymerize. Ultrasections of approximately 60 nm were placed on 100 mesh Copper grids (EMS #G100H-Cu) and stained with 2% uranyl acetate (EMS # 22400) and lead citrate (VWR #1.07398). Grids were imaged using a Jeol JEM-1230 transmission electron microscope at 80 kV. Different organelles were counted in images acquired at 20.000× magnification from the cytoplasm in hepatocytes, in liver samples of both *WT* and *Pah-R261Q* mice (40 TEM micrographs for each mouse).

For protein TEM with negative staining, 5 µl samples of protein solutions (20 µg/ml) in the indicated buffer were allowed to be absorbed (1 min) on a glow discharged, 300 mesh (EMS # G300H-Cu), carbon-coated grid. The grids were then stained for 1 min in 2% uranyl acetate. Grids were allowed to dry for 30 min before imaging. The incorporation of two consecutive washing steps with double distilled water after absorption of the protein improved the background, but we observed loss of aggregates.

### Oxidative stress assay (Trolox)

The antioxidant capacity of small molecules (such as ascorbate, glutathione, and vitamin E) in *Pah-R261Q* and WT liver homogenates was determined by the Trolox equivalent antioxidant capacity assay using the colorimetric Total Antioxidant Capacity Assay kit (Abcam). Samples were diluted 1:1 with the included reagent Protein Mask to avoid the potential contribution of other biological species. Otherwise, the standard protocol indicated in the product manual was followed. Results were obtained by interpolation to a Trolox (reference antioxidant) standard curve and expressed as Trolox equivalent antioxidant capacity.

### Gene (mRNA) expression

Total RNA was extracted from powdered livers of *Pah-R261Q* and *WT* mice (3-months-old; *n* = 5–6 per group) with the QIAmp RNA Blood Mini Kit (QIAGEN) and translated into cDNA using the Reverse Transcription System (Promega). Quantitative PCR was performed in standard triplicate assays for each mouse sample with 50 ng of cDNA using TaqMan technology, an ABI Prism 7700 sequence detector, and the TaqMan Universal PCR Master Mix from ABI. Detailed information about the specific transcript detection cannot be given, as we used ABI TaqMan Gene Expression Assays, which are under a proprietary license, and the exact primer and probe sequence are not disclosed. The ABI assay numbers for the indicated murine mRNA and NCBI nucleotide sequence numbers are summarized in Supplementary Table 4. Murine *Gapdh*-mRNA was included as a control for normalization and the analyses of the relative gene expression were performed based on the 2^−ΔΔCt^ method^77^.

### Software

The computer algorithm TANGO^47^ was used for the prediction of aggregating regions in the relevant mutants and WT PAH sequences. Calculations were performed online (http://tango.crg.es/) with default parameters. PAH particles were analyzed and measured using Fiji ImageJ.

### Statistics and reproducibility

Quantitative data are presented as mean ± standard deviation (SD). Individual values are plotted as circles in both scatter dot plots and bar graphs, except in the inset of Fig. 1e where the circles represent the mean for the group for each time point. Statistical significant differences were determined by unpaired two-tailed *t* test for pairs of groups, except when indicated, i.e., two-tailed Mann Whitney *U* tests were used for the analysis of data in Table 1 and Supplementary Table 2. For comparisons of more than two groups one-way ANOVA followed by post hoc Tukey test was used, except for data in the inset of Supplementary Fig. 4 where due to unequal variance and sample size Brown–Forsythe and Welch ANOVA test was used followed by Dunnett’s multiple comparisons test, as indicated. Note that despite the fact that mean ± SD is presented in this figure, the analysis is run on mean AUC ± SEM data, which is also the case for the *t* test applied on the AUC data in the inset of Fig. 1d and inset of Fig. 2c. We considered *p* < 0.05 as statistically significant and *p* values are provided with accurate numbers down to <0.0001. Statistical analyses were performed using GraphPad Prism^™^ (version 8.3.0, San Diego) software and the applied analysis in each case is included in the corresponding Table and Figure legend only for the results for which statistical significance was obtained.

For representations of quantitative data, *n* customarily refers to the number of independent biological samples (mice) examined in each analysis, except when explicitly referring to the number of independent enzyme purifications. When required, i.e., Figure 5c and Supplementary Fig. 7, the number of sections and/or particles analyzed is also provided in the corresponding figure legend.

The representative Western blots (Fig. 3a, b) have been repeated at least three times in independent experiments, using different mice in each experiment (*n* ≥ 3 for each genotype). Densitometric results obtained with the Western blots are represented in Fig. 3c. The agarose gel in Supplementary Fig. 2c represents the procedure used for genotyping after breeding and similar results have been obtained at least 20 times in independent experiments including in total *n* ≥ 40 mice of each genotype. Representative micrographs showing results from immunofluorescence and IHC (Figs. 4, 5a,b and 6a,b) have been successfully repeated with *n* ≥ 3 for each genotype in independent experiments. The results in the representative micrograph from TEM with purified p.R261Q-PAH (Supplementary Fig. 6b) have been observed with *n* = 3 protein preparations. For the TEM of hepatic tissue, representative micrographs with the relevant organelles are shown and have been observed in liver samples from *n* = 3 mice in each genotypic group.

### Ethical compliance

We have complied with all relevant ethical regulations for mice breeding, testing and research. The animal experiments in this study have received the appropriate approval from the Norwegian Food Safety Authority (Brumunddal, Norway) (approved application 20168698) and performed at the Laboratory Animal Facility, University of Bergen, according to the guidelines and standards from the Regulation on the use of animals in the research of this institution.

### Reporting summary

Further information on research design is available in the Nature Research Reporting Summary linked to this article.

## Supplementary information

Supplementary Information

Reporting Summary

## Data Availability

Source data underlying Figs. 1–6, Supplementary Figs. 2c and 3–6, Tables 1 and 2 and Supplementary Tables 2 and 3 are provided with this paper as a Source Data file. The rest of the data that support Supplementary Figs. 7 and 8 within this paper, other findings and the mouse and derived materials will be made available upon reasonable request to the correspondence author. Web-links for NCBI nucleotide sequences: *Pah–mRNA* NM_008777.3 https://www.ncbi.nlm.nih.gov/nuccore/NM_008777.3, *Hsc70–mRNA* NM_031165.4 https://www.ncbi.nlm.nih.gov/nuccore/NM_031165.4, *Gch1–mRNA* NM_008102.3 https://www.ncbi.nlm.nih.gov/nuccore/NM_008102.3, *Hsf1–mRNA* NM_008296.2 https://www.ncbi.nlm.nih.gov/nuccore/NM_008296.2, *stub1–mRNA* NM_019719.3 https://www.ncbi.nlm.nih.gov/nuccore/NM_019719.3, *Gchfr –mRNA* NM_177157.4 https://www.ncbi.nlm.nih.gov/nuccore/NM_177157.4, *Dnajc12–mRNA* NM_001253685.1 https://www.ncbi.nlm.nih.gov/nuccore/NM_001253685.1, *p62-mRNA* NM_001290769.1 https://www.ncbi.nlm.nih.gov/nuccore/NM_001290769.1, *Hsp70 –mRNA* NM_001163434.1 https://www.ncbi.nlm.nih.gov/nuccore/NM_001163434.1, *Ap-1–mRNA* NM_001243043.1 https://www.ncbi.nlm.nih.gov/nuccore/NM_001243043.1, *Gapdh*-mRNA NM_008084.3 https://www.ncbi.nlm.nih.gov/nuccore/NM_008084.3 Web-link for PAH structure in complex with BH_4_ PDB 6HYC (https://www.rcsb.org/structure/6HYC) Web-link for data on C57BL/6J mouse strain (https://www.jax.org/strain/000664). Source data are provided with this paper.

## Source data

Source Data
