# Supplementary material for: The Pah-R261Q mouse reveals oxidative stress associated with amyloid-like hepatic aggregation of mutant phenylalanine hydroxylase
Source: Nat Commun. 2021 Apr 6;12:2073. doi: 10.1038/s41467-021-22107-1 (PMC8024259; doi:10.1038/s41467-021-22107-1)
Supplement: Supplementary file 1 — Supplementary Information [file 41467_2021_22107_MOESM1_ESM.pdf]

**Supplementary Table 1. Mouse genome off-target analysis.** The 16 possible off-target loci selected from BLAST analysis of the guide RNA towards the *Mus musculus* GRCm38/mm10 assembly genome and assayed by heteroduplex mobility. No off-target interactions could be detected.

| Chromosome (No.) | DNA sequence[1] (Non-seed_Seed_PAM) | Mismatches (No.) | Target site (Name) |
|---|---|---|---|
| 7 | AGgGGAAG_ACTCaGAAGGCC_AGG | 2 | Overlap exon *Shisa7* |
| | | | Overlap exon *C920025E04Rik* |
| 17 | AGcGGgAG_ACTCGGAAaGCC_AGG | 3 | Overlap exon *Ptk7* |
| 15 | tGTaGAAG_ACTCGGAAGtCC_CGG | 3 | Overlap intron *Nell2* |
| 3 | tGTGcAgG_ACTgGGAAGGCC_AAG | 4 | Overlap exon *RP24-275F18.4* |
| 5 | taTcGAAG_ACTCGGAAGGaC_AGG | 4 | Overlap exon *Pcolce* |
| 5 | ttTGGAgG_ACTCGGAgGGCC_CGG | 4 | Overlap exon *Cyth3* |
| 11 | AGgGagAG_ACTgGGAAGGCC_TGG | 4 | Boundary exon *Flt4* |
| 10 | tGTctAAG_ACTCaGAAGGCC_AGG | 4 | Overlap intron *Stat6* |
| 11 | tcTaGAAG_cCTCGGAAGGCC_AAG | 4 | Overlap intron *Ntn1* |
| 11 | AtTccAAG_ACTCGGAAGGCt-GAG | 4 | Overlap intron *Fat2* |
| 14 | cacGGAAG_ACTCGGAAGGCt-GAG | 4 | Overlap intron *Arhgap22* |
| 16 | ccTcGAAG_ACTCGGAAGaCC-TGG | 4 | Overlap intron *Lpp* |
| 6 | AGTcGgAa_ACTCGGAAGaCC-AGG | 4 | Overlap intron *Plxna4* |
| 6 | AGTGGcgc_ACTCGGAAGGCa-GAG | 4 | Overlap intron *Raf1* |
| 8 | gGTGGcAa_ACTCGGAAGtCC-AAG | 4 | Overlap intron *Gm20388* |

[1]The DNA sequence is divided, for clarity purposes, in the non-seed region, seed region, and protospaceradjacent motif (PAM), with lowercases indicating the mismatched nucleotides.

**Supplementary Table 2. Complete list of metabolites analyzed in isolated serum samples.** The serum levels of 72 metabolites were analyzed by chromatographic separation and mass spectrometry detection.

| Metabolite (Name) | *Pah-WT* (μM) | *Pah-R261Q* (μM) | *p*-value[1] | Method |
|---|---|---|---|---|
| Total Homocysteine | 5.23 (2.41) | 6.23 (2.37) | 0.284 | GC-MS/MS[a] |
| Methylmalonic acid | 0.701 (0.095) | 0.585 (0.118) | 0.060 | |
| Total cysteine | 241 (48) | 267 (35) | 0.708 | |
| Methionine | 61.8 (9.9) | 57.8 (11.4) | 0.172 | |
| Serine | 144 (28) | 136 (21) | 0.123 | |
| Glycine | 318 (55) | 293 (45) | 0.354 | |
| Cystathionine | 1.04 (0.57) | 1.10 (0.31) | 0.686 | |
| Kynurenine | 0.740 (0.234) | 0.573 (0.264) | 0.080 | |
| Sarcosine | 1.29 (0.59) | 0.958 (0.414) | 0.234 | |
| Histidine | 70.0 (10.5) | 71.1 (11.5) | 0.686 | |
| **Tryptophan** | **103 (24)** | **82.9 (30.9)** | **0.013** | |
| Ornithine | 61.2 (16.3) | 58.9 (12.3) | 0.624 | |
| **Aspartic acid** | **27.4 (14.4)** | **22.7 (11.0)** | **0.043** | |
| **Glutamic acid** | **39.5 (20.7)** | **29.4 10.2)** | **0.008** | |
| Lysine | 306 (46) | 279 (45) | 0.470 | |
| **Alanine** | **444 (71)** | **362 (80)** | **0.010** | |
| **Phenylalanine** | **71.9 (10.3)** | **113 (22)** | **0.0000004** | |
| Isoleucine | 87.5 (12.8) | 101 (24) | 0.091 | |
| Leucine | 143 (23) | 164 (42) | 0.075 | |
| Proline | 90.2 (25.4) | 73.0 (17.2) | 0.085 | |
| Valine | 220 (28) | 233 (38) | 0.563 | |
| Asparagine | 49.0 (13.0) | 47.9 (8.8) | 0.103 | |
| **Glutamine** | **687 (76)** | **622 (112)** | **0.043** | |
| Threonine | 145 (29) | 140 (30) | 0.123 | |
| **Tyrosine** | **81.3 (25.3)** | **77.9 (14.0)** | **0.050** | |
| **α-Ketoglutaric acid** | **38.6 (15.2)** | **26.3 (10.8)** | **0.003** | |
| 3-Hydroxyisobutyrate | 22.8 (10.6) | 22.1 (10.0) | 0.773 | |
| 2-Hydroxybutyrate | 18.3 (6.4) | 14.1 (6.4) | 0.529 | |
| **β-Hydroxybutyrate** | **150 (99)** | **282 (133)** | **0.029** | |
| Acetoacetate | 20.6 (21.7) | 28.9 (15.9) | 0.579 | |
| Choline | 25.7 (9.3) | 30.1 (7.4) | 0.236 | LC-MS/MS[b] |
| Betaine | 67.3 (39.9) | 69.2 (16.8) | 0.808 | |
| Dimethylglycine | 9.70 (4.32) | 10.6 (2.2) | 0.715 | |
| Creatinine | 8.95 (2.29) | 9.24 (2.35) | 0.648 | |
| Methionine sulfoxide | 2.34 (1.29) | 1.92 (1.20) | 0.191 | |
| Arginine | 115 (17) | 112 (19) | 0.574 | |
| Asymm.dimethylarginine | 0.759 (0.244) | 0.872 (0.218) | 0.855 | |
| Symm.dimethylariginine | 0.296 (0.075) | 0.360 (0.087) | 0.202 | |
| Homoarginine | 0.927 (0.207) | 0.783 (0.277) | 0.403 | |
| **Trimethyllysine** | **0.803 (0.165)** | **0.976 (0.213)** | **0.050** | |
| Trimethylamineoxide | 3.57 (6.31) | 2.86 (6.02) | 0.599 | |
| **Creatine** | **154 (40)** | **127 (30)** | **0.026** | |

| | | | | |
|---|---|---|---|---|
| 1-Methylhistidine | 2.90 (0.43) | 2.70 (0.66) | 0.976 | |
| 3-Methylhistidine | 4.04 (1.50) | 5.03 (2.12) | 0.354 | |
| 5-Methyltetrahydrofolate | 131 (23) | 119 (33) | 0.406 | |
| 5-Formyltetrahydrofolate | n.d. | n.d. | ---- | |
| Folic acid | 1.39 (1.24) | 1.32 (0.93) | 0.908 | |
| 4-α-Hydroxy-5-methyl-THF | 6.05 (1.53) | 5.53 (2.65) | 0.684 | LC-MS/MS[c] |
| *p*-Aminobenzoylglutamate | 35.1 (17.3) | 32.5 (16.4) | 0.853 | |
| Acetoamidobenzoylglutamate | 7.43 (2.96) | 6.73 (3.00) | 0.853 | |
| Pyridoxal 5'-phosphate | 0.232 (0.096) | 0.276 (0.050) | 0.351 | |
| Pyridoxal | 0.223 (0.085) | 0.217 (0.102) | 0.958 | |
| 4-Pyridoxic acid | 0.034 (0.09) | 0.036 (0.021) | 0.681 | |
| Pyridoxine | n.d. | n.d. | ---- | |
| Thiamine | 0.331 (0.028) | 0.273 (0.058) | 0.112 | |
| Thiamine monophosphate | 0.372 (0.100) | 0.425 (0.082) | 0.758 | |
| Riboflavin | 0.066 (0.007) | 0.066 (0.013) | 0.918 | |
| Flavin mononucleotide | 0.037 (0.004) | 0.032 (0.002) | 0.252 | |
| Neopterin | 0.002 (0.001) | 0.002 (0.001) | 0.138 | |
| Cotinine | n.d. | n.d. | ---- | |
| Trans-3'-hydroxycotinine | n.d. | n.d. | ---- | |
| 3-Hydroxykynurenine | 0.056 (0.019) | 0.063 (0.017) | 0.408 | LC-MS/MS[d] |
| Kynurenic acid | 0.050 (0.021) | 0.058 (0.013) | 0.351 | |
| Xanthurenic acid | 0.030 (0.024) | 0.047 (0.019) | 0.606 | |
| Anthranilic acid | 0.030 (0.005) | 0.029 (0.006) | 0.837 | |
| 3-Hydroxyanthranilic acid | 0.012 (0.007) | 0.012 (0.004) | 0.606 | |
| Picolinic acid | 0.108 (0.056) | 0.146 (0.058) | 0.791 | |
| **Quinolinic acid** | **0.178 (0.090)** | **0.130 (0.041)** | **0.023** | |
| Nicotinic acid | n.d. | n.d. | ---- | |
| Nicotinamide | 4.74 (0.74) | 4.29 (1.11) | 0.351 | |
| N1-methylnicotinamide | 0.314 (0.066) | 0.251 (0.140) | 0.596 | |
| Trigonelline | 2.21 (1.09) | 1.49 (1.28) | 0.351 | |

Concentrations are expressed as arithmetic mean ± SD; $n = 19$ *WT* and 19 *Pah-R261Q* mice. n.d., non-detectable (below detection limit).

[1]Two-tailed *p*-values for differences between serum concentration in *Pah-WT* and *Pah-R261Q* from Mann Whitney U test. The highlighted metabolites in bold text show $p \leq 0.5$.

**References**


a. Midttun O*, et al.* Combined Measurement of 6 Fat-Soluble Vitamins and 26 Water-Soluble Functional Vitamin Markers and Amino Acids in 50 muL of Serum or Plasma by High-Throughput Mass Spectrometry. *Anal Chem* **88**, 10427-10436 (2016).
b. Midttun O, Kvalheim G, Ueland PM. High-throughput, low-volume, multianalyte quantification of plasma metabolites related to one-carbon metabolism using HPLC-MS/MS. *Anal Bioanal Chem* **405**, 2009-2017 (2013).
c. Hannisdal R, Ueland PM, Svardal A. Liquid chromatography-tandem mass spectrometry analysis of folate and folate catabolites in human serum. *Clin Chem* **55**, 1147-1154 (2009).
d. Midttun O, Hustad S, Ueland PM. Quantitative profiling of biomarkers related to B-vitamin status, tryptophan metabolism and inflammation in human plasma by liquid chromatography/tandem mass spectrometry. *Rapid Commun Mass Spectrom* **23**, 1371-1379 (2009).

**Supplementary Table 3. Aromatic amino acid levels in brain**

| Amino acid | *Pah-* | *n*[1] | Brain (nmol/mg protein) |
|---|---|---|---|
| L-Phe | *WT* | 6 | 0.38 ± 0.04 |
| | *R261Q* | 5 | 0.42 ± 0.12 |
| L-Tyr | *WT* | 6 | 0.29 ± 0.10 |
| | *R261Q* | 5 | 0.31 ± 0.09 |
| L-Trp | *WT* | 6 | 0.13 ± 0.02 |
| | *R261Q* | 5 | 0.13 ± 0.02 |

[1]*n*, number of mice analysed in each group (3-month-old).

**Supplementary Table 4. List of probes and primers used in the study**

***Quantitative RT-PCR probes***

| Name | Assay ID | chromosome location | Nucleotide sequence accession code |
|---|---|---|---|
| mouse phenylalanine hydroxylase (PAH) | Mm00500918_m1 | Chr.10: 87521795 - 87584137 on Build GRCm38 | NM_008777.3 |
| mouse Hsc70 (Hspa8) | Mm01731394_gH | Chr.9: 40801273 - 40805199 on Build GRCm38 | NM_031165.4 |
| mouse GTP cyclohydrolase 1 (Gch1) | Mm01322973_m1 | Chr.14: 47153895 - 47189402 on Build GRCm38 | NM_008102.3 |
| mouse heat shock factor 1 (Hsf1) | Mm01201402_m1 | Chr.15: 76477395 - 76500978 on Build GRCm38 | NM_008296.2 |
| mouse STIP1 homology and U-box containing protein 1 (Stub1) | Mm00490634_m1 | Chr.17: 25830636 - 25833361 on Build GRCm38 | NM_019719.3 |
| mouse GTP cyclohydrolase I feedback regulator (Gchfr) | Mm00622819_m1 | Chr.2: 119167788 - 119172389 on Build GRCm38 | NM_177157.4 |
| Mouse DNAJC12 | Mm00497038_m1 | Chr.10: 63382443 - 63408840 on Build GRCm38 | NM_001253685.1 |
| mouse sequestosome 1 (p62) | Mm00448091_m1 | Chr.11: 50200152 - 50210820 on Build GRCm38 | NM_001290769.1 |
| mouse HSP70 (Hspa5) | Mm00517691_m1 | Chr.2: 34772090 - 34776529 on Build GRCm38 | NM_001163434.1 |
| mouse adaptor protein complex AP-1, beta 1 subunit (Ap1b1) | Mm01187764_m1 | Chr.11: 4947521 - 5042794 on build GRCm38 | NM_001243043.1 |
| mouse glyceraldehyde-3-phosphate dehydrogenase (GAPDH) | Mm99999915_g1 | Chr.6: 125161338 - 125166511 on build GRCm38 | NM_008084.3 |

***Primers for genotyping***

| Name | Primer sequence |
|---|---|
| PAH genotype forward primer | 5’-ATGCAGGATATCTAAGGTGCC-3’ |
| PAH genotype reverse primer | 5’-GAGATGCTGAGATCACTTGGC-3’ |

## Supplementary Fig. S1

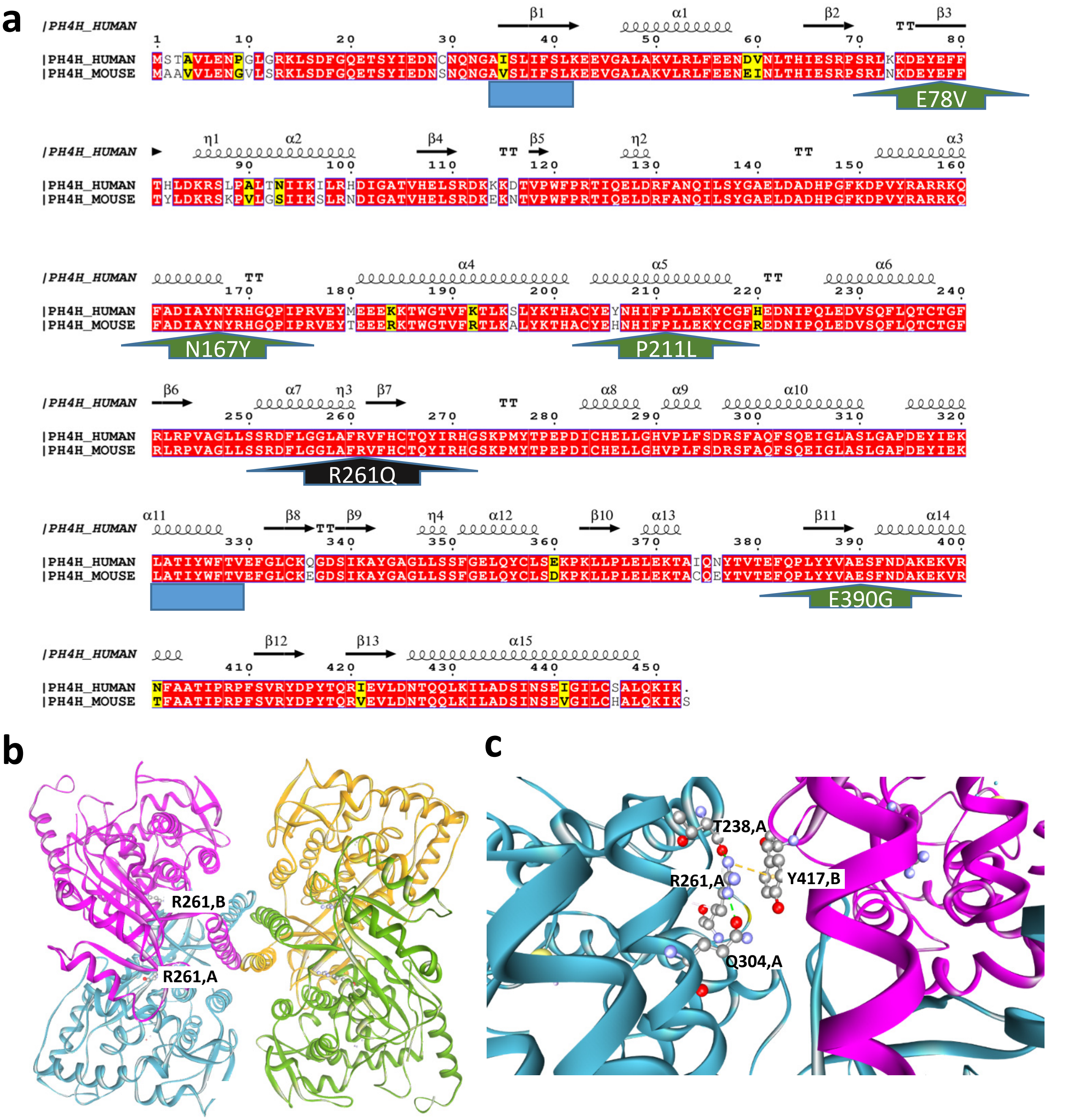


**Supplementary Fig. 1. Sequence comparison and structural representation of PAH.** a) Sequence alignment of human (P000439) and mouse (P16331) PAH. Identical residues (red background), conservative mutations (yellow background) and non-conservative mutations (white background) are highlighted. Prepared with ESPript (http://espript.ibcp.fr) with secondary structural motifs on top extracted from tetrameric human PAH (PDB 6HYC). Motifs predicted by TANGO (http://tango.crg.es/) to have high propensity to aggregate in a β-cross manner in the WT sequence are pointed by blue boxes, and motifs appearing in specific PKU mutant sequences are shown as broad green arrows, or as a black arrow for the R261→Q mutation studied here. b) Structural location of Arg261 in PAH subunits A (R261,A) and B (R261,B) (PDB 6HYC); each subunit is shown in a different color. c) Interactions of R261,A intra-subunit (with T238,A and Q304,A) and inter-subunit within one dimer (with Y417,B). The highlighted residues are shown in ball and stick representation.

**Supplementary Fig. S2**

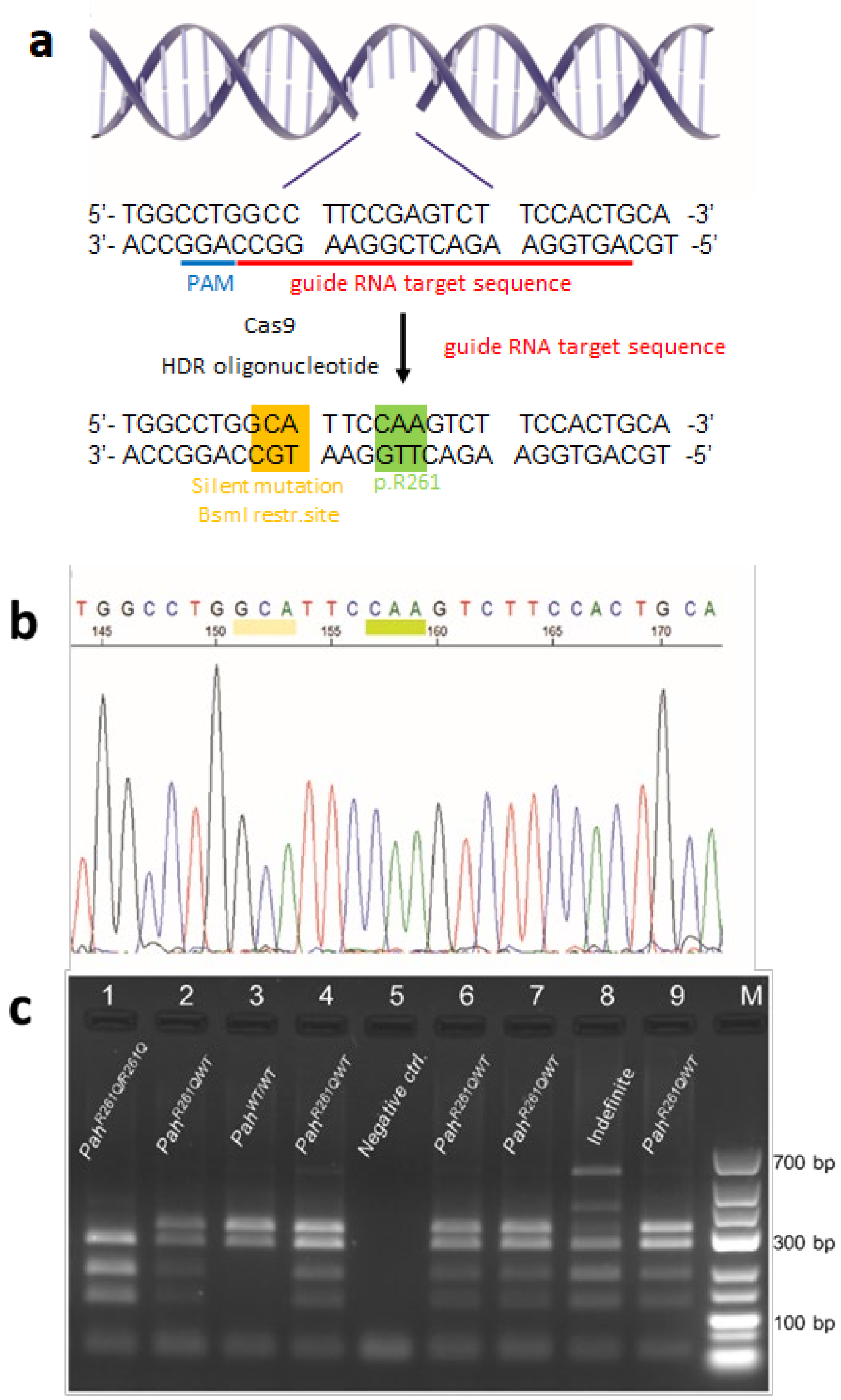


**Supplementary Fig. 2. Generation and genotyping of the novel *Pah-R261Q* mouse model.** a) Graphic schematic illustration of the CRISPR/Cas9 targeting strategy. The guide RNA target sequence (in red) and "PAM" (in blue) interact, complementarily, with the region of interest in exon 7 of *Pah* gene on murine chromosome 10 (NM_008777.3). The two introduced point mutations c.777C>A and c.782G>A (p.Arg261Gln) provided a successful Cas9 endonuclease action and homology-directed repair (HDR) integration, and are highlighted in ochre and green background, respectively. b) Confirmation of the germline-transmitted *Pah* mutation by evaluation of sequence chromatogram. c) Representative example of an agarose gel from a genotyping experiment. The encountered genotypes were $Pah^{WT/WT}$ (2 bands), $Pah^{R261Q/R261Q}$ (3 bands) and $Pah^{R261Q/WT}$ (4 bands); molecular weights provided in main text. M, DNA ladder; negative ctrl., ddH$_2$0 sample. For c) Source data are provided as a Source Data file

**Supplementary Fig. S3**

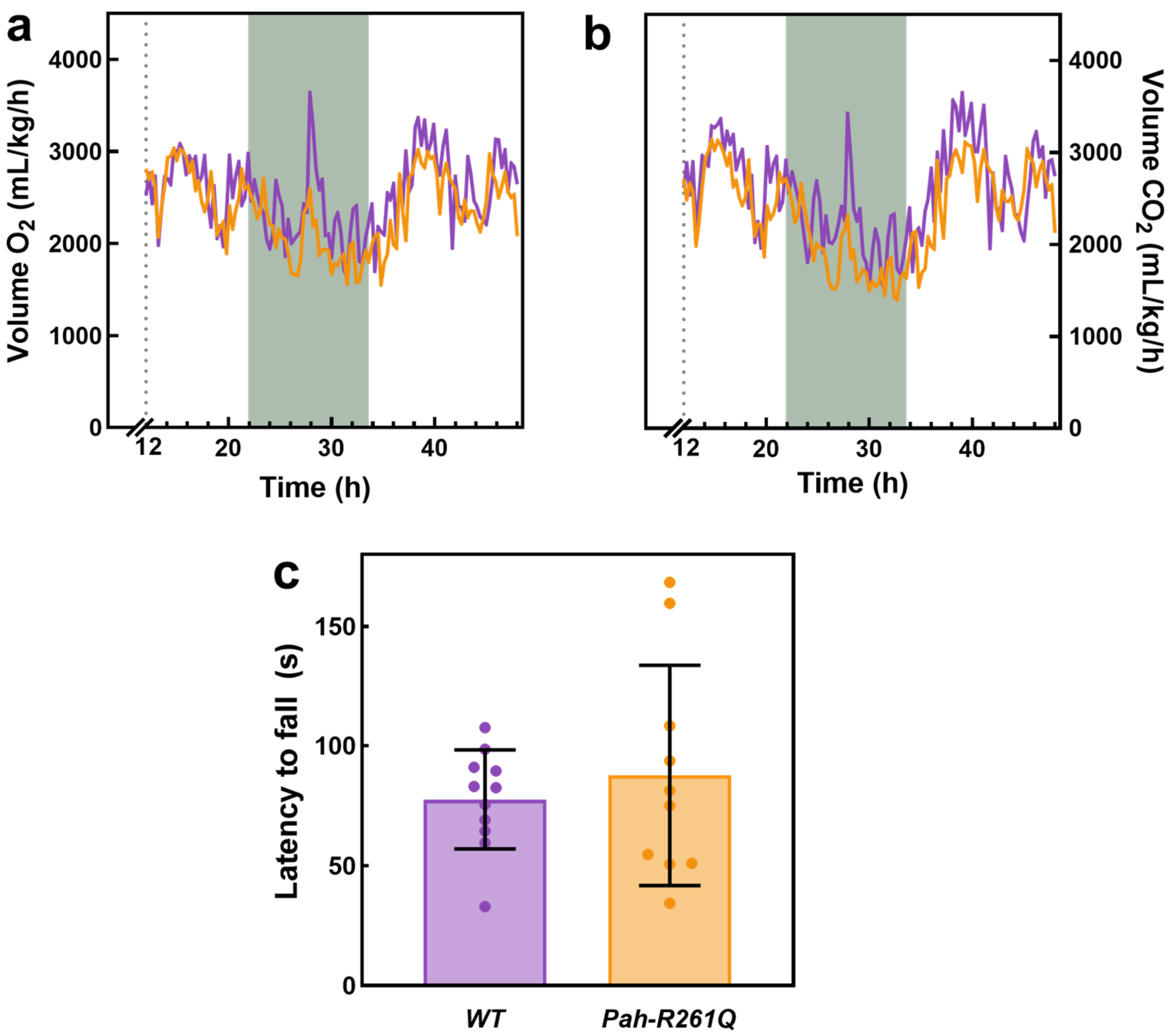


**Supplementary Fig. 3. Time course of the volume of $O_2$ consumed and $CO_2$ produced and rotarod performance test**. Volume of $O_2$ consumed (a) and $CO_2$ produced (b), measured in the metabolic cage. Averaged recordings with $n = 3$ *WT* and 5 *Pah-R261Q* mice in independent experiments, with one mouse per cage and 121 observations/animal. The 12 h acclimatization period was not included in the recordings. c) Motor function assessment by rotarod test. The bars express mean ± SD ($n$= 11 *WT* and 10 *Pah-R261Q* mice, analyzed independently). The circles represent individual values for each mouse. In all panels, the data for *WT* are depicted in purple and *Pah-R261Q* in ochre. Source data are provided as a Source Data file.

**Supplementary Fig. S4**

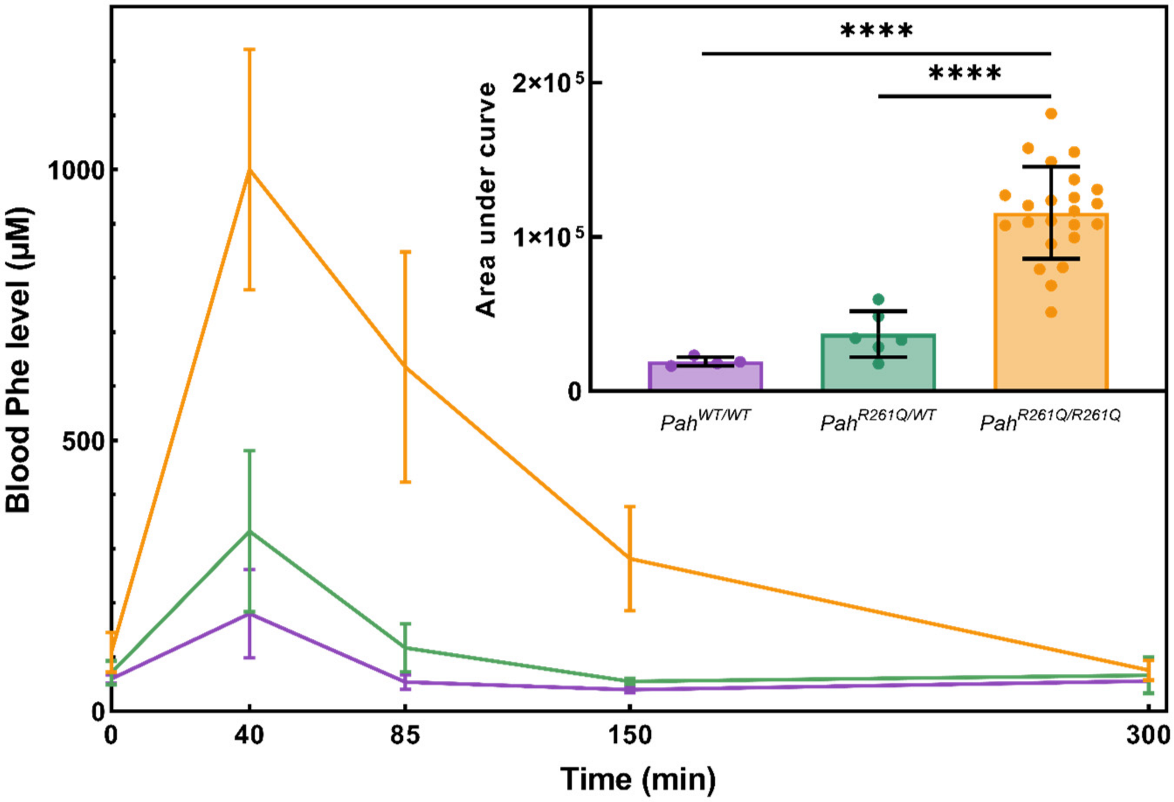


**Supplementary Fig. 4. Effect of Phe challenge on blood L-Phe concentration in mice with different genotypes.** At time 0, an L-Phe challenge (200 µg/g body weight) was provided by i.p. to *WT* (purple)*,* $Pah^{R261Q/WT}$ (green) *and* $Pah^{R261Q/R261Q}$ (ochre) mice. The results represent the mean ± SD (*n*, number of animals, was *4* $Pah^{WT/WT}$, 6 $Pah^{R261Q/WT}$ and 23 $Pah^{R261/R261Q}$ mice). Inset, area under the curve (AUC) for the time dependence of L-Phe concentration between 0 and 300 min for the three genotypic groups. Data are presented as mean AUC ± SD. The circles represent the individual values for each mouse. Statistical significance between the groups was analyzed by Brown-Forsythe and Welch ANOVA test followed by Dunnett`s multiple comparison test, providing $p$ <0.0001 (****) for both $Pah^{R261/R261Q}$ *vs.* $Pah^{WT/WT}$ and $Pah^{R261/R261Q}$ *vs.* $Pah^{R261/WT}$. Source data are provided as a Source Data file.

**Supplementary Fig. S5**

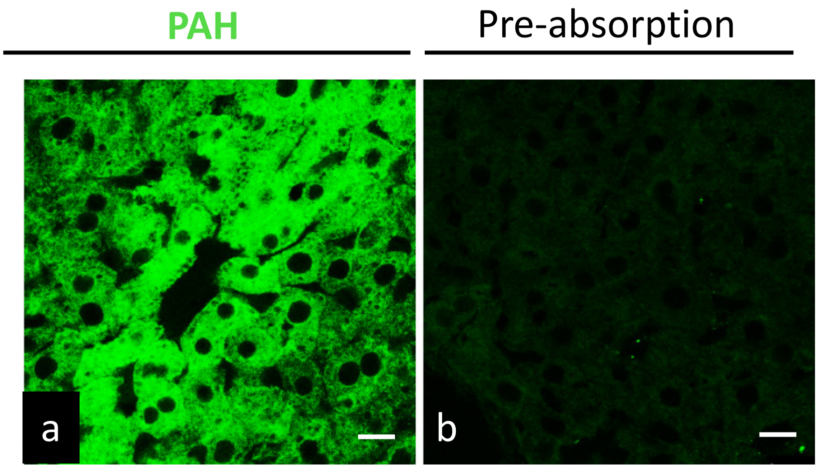


**Supplementary Fig. 5. PAH immunostaining in liver of *WT* mice; specificity of the PAH antibody.** a) Immunofluorescence staining reveals PAH immunoreactivity in the liver of *WT* mice. b) Disappearance of PAH staining in the *WT* liver after an antibody pre-absorption treatment with purified recombinant PAH. Scale bars are 10 µm. These are representative images based on experiments performed with two liver lysates, prepared from a different *WT* mouse each ($n = 2$), with three replicates for each preparation. Source data are provided as a Source Data file.

**Supplementary Fig. S6**

a

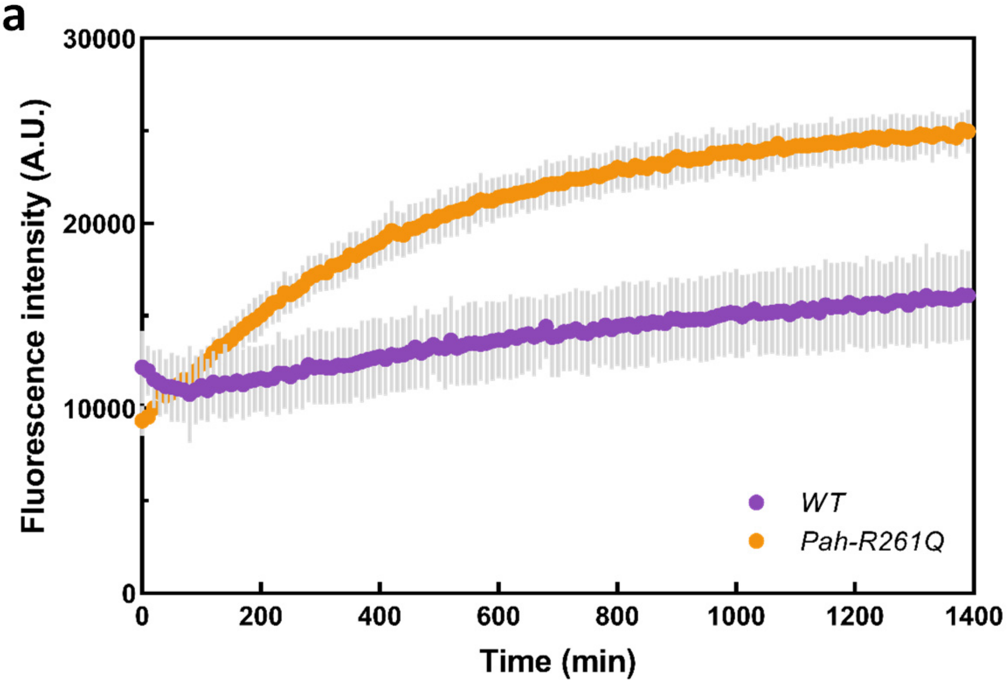


b

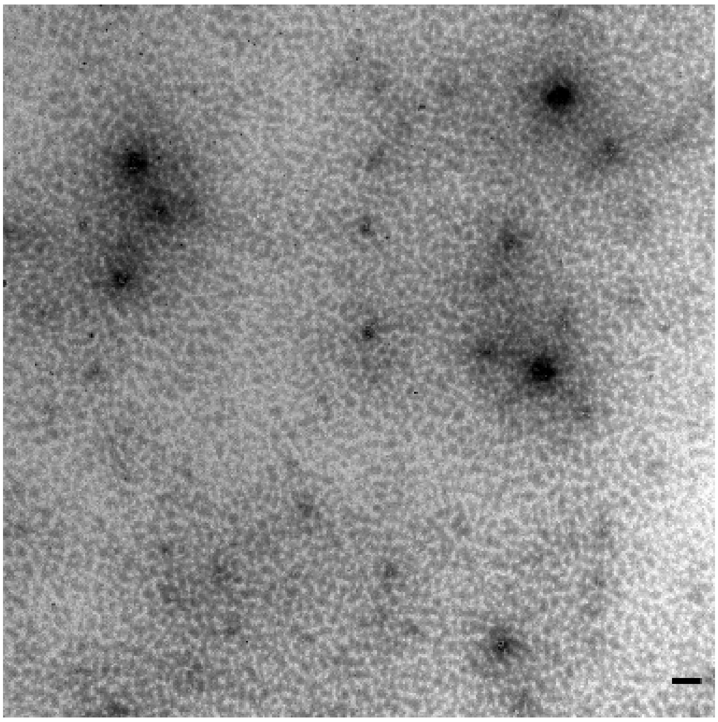

Scale bar 100 nm

**Supplementary Fig. 6. Amyloid-like aggregation of the p.R261Q-PAH protein.** a) Amytracker™680 assay with recombinant purified proteins WT-PAH (purple) and p.R261Q-PAH (ochre), with 1 mg/ml of each protein in 20 mM Na-Hepes, 200 mM NaCl, pH 7 and incubation at 37 $^{o}$C. The results represent the mean and SD (gray lines) at each time point for $n$ =3 (independent protein samples). b) Transmission electron microscopy (TEM) with negative staining for p.R261Q-PAH, after 5 h incubation at 37 $^{o}$C and buffer exchange to 20 mM Na-phosphate, pH 7. The protein was applied to the grid at 0.02 mg/ml; at time 0, no particles > 20 nm in diameter are observed. Representative TEM micrograph from $n$ =3 (independent protein samples). Tetrameric PAH has a diameter of 10 nm (PDB 6HYC). Source data are provided as a Source Data file.

**Supplementary Fig. S7**

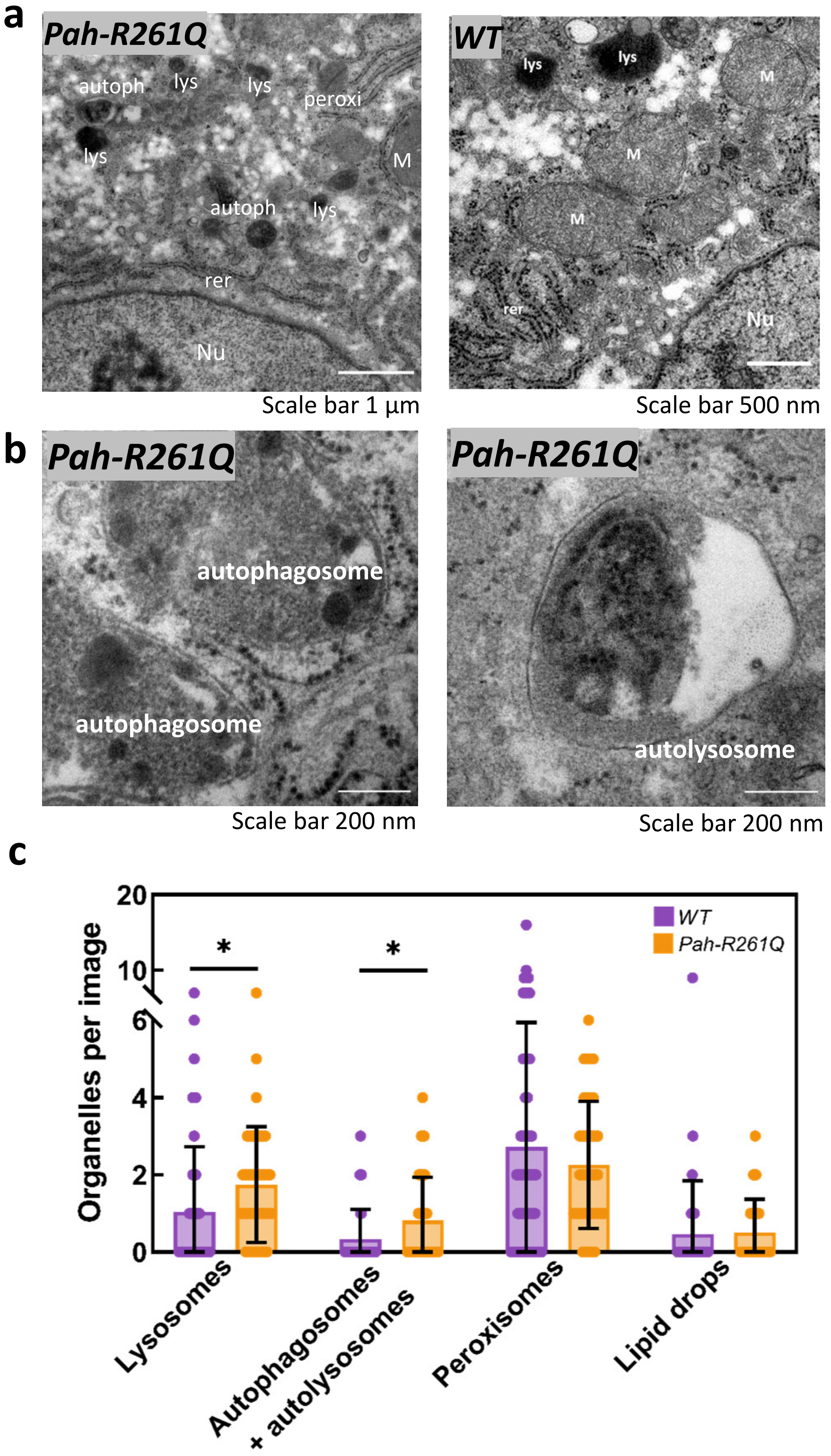


**Supplementary Fig. 7. Transmission electron microscopy (TEM) of liver tissue.** a) Representative micrographs showing normal cellular and organelle morphology in *Pah-R261Q* mice. Nu, nucleus; M, mitochondria; lys, lysosome; autoph, autophagosome; peroxi, peroxisomes; rer, rough endoplasmic reticulum. b) TEM micrographs showing two double-membrane autophagosomes and one autolysosome, representative of $n$= 3 biological replicates per mice group. c) Quantification of different organelles in images acquired in hepatocytes from both *WT* (purple) and *Pah-R261Q* (ochre) mice. 40 TEM images at 20.000 magnification were analyzed for each mice. The results represent the mean ± SD. Statistical significance between both groups was calculated using two-tailed unpaired t-test; $p$ = 0.0402 for lysosomes (*) and $p$ = 0.0151 (*) for autophagosomes + autolysosomes.

**Supplementary Fig. S8**

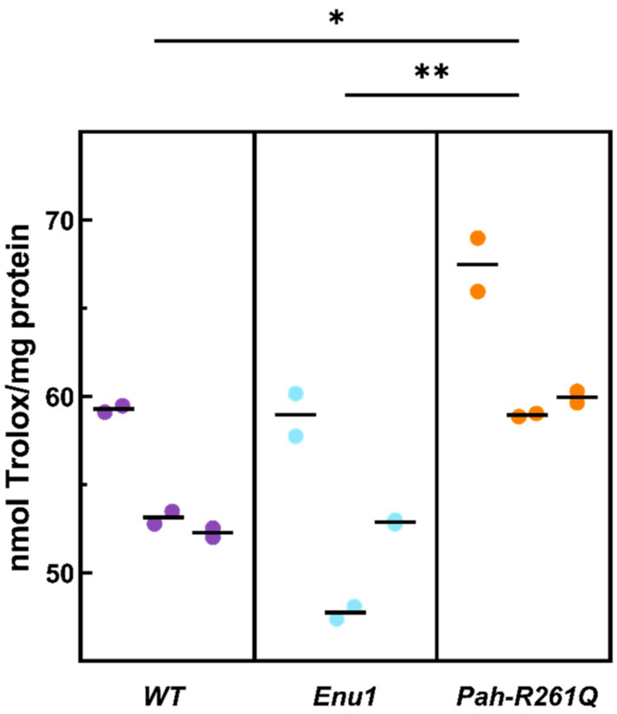


**Supplementary Fig. 8. Measurement of oxidative stress in liver lysates of mice models with different genotype.** Total antioxidant capacity using the Trolox (6-hydroxy-2,5,7,8-tetramethylchroman-2-carboxylic acid) assay in liver lysates of *WT* (purple), *Pah*$^{R261Q/WT}$ (blue) *and Pah*$^{R261Q/R261Q}$ (ochre). Two independent lysates per mouse and $n$ = 3 (biological replicates) per mice group were analyzed. Data are presented as means, and circles representing individual values, for each lysate. Statistical significance between the groups was calculated by one-way ANOVA followed by post hoc Tukey test, providing $p$ = 0.029 (*) for *Pah-R261Q* vs. *WT* (*) and $p$ = 0.006 (**) for *Pah-R261Q* vs. *Enu1*.
