# Supplementary material for: The Pah-R261Q mouse reveals oxidative stress associated with amyloid-like hepatic aggregation of mutant phenylalanine hydroxylase
Source: Nat Commun. 2021 Apr 6;12:2073. doi: 10.1038/s41467-021-22107-1 (PMC8024259; doi:10.1038/s41467-021-22107-1)
Supplement: Supplementary file 2 — Reporting Summary [file 41467_2021_22107_MOESM2_ESM.pdf]

nature research

Corresponding author(s): Aurora Martinez

Last updated by author(s): Jan 1, 2021

Reporting Summary

Nature Research wishes to improve the reproducibility of the work that we publish. This form provides structure for consistency and transparency in reporting. For further information on Nature Research policies, see Authors & Referees and the Editorial Policy Checklist .

Statistics

For all statistical analyses, confirm that the following items are present in the figure legend, table legend, main text, or Methods section.

n/a	Confirmed	
☐	☒	The exact sample size (*n*) for each experimental group/condition, given as a discrete number and unit of measurement
☐	☒	A statement on whether measurements were taken from distinct samples or whether the same sample was measured repeatedly
☐	☒	The statistical test(s) used AND whether they are one- or two-sided<br>*Only common tests should be described solely by name; describe more complex techniques in the Methods section.*
☒	☐	A description of all covariates tested
☐	☒	A description of any assumptions or corrections, such as tests of normality and adjustment for multiple comparisons
☐	☒	A full description of the statistical parameters including central tendency (e.g. means) or other basic estimates (e.g. regression coefficient) AND variation (e.g. standard deviation) or associated estimates of uncertainty (e.g. confidence intervals)
☐	☒	For null hypothesis testing, the test statistic (e.g. *F*, *t*, *r*) with confidence intervals, effect sizes, degrees of freedom and *P* value noted<br>*Give P values as exact values whenever suitable.*
☒	☐	For Bayesian analysis, information on the choice of priors and Markov chain Monte Carlo settings
☒	☐	For hierarchical and complex designs, identification of the appropriate level for tests and full reporting of outcomes
☒	☐	Estimates of effect sizes (e.g. Cohen's *d*, Pearson's *r*), indicating how they were calculated

Our web collection on statistics for biologists contains articles on many of the points above.

Software and code

Policy information about availability of computer code

Data collection	Only commercial software has been used: Oxymax v.5.09 and CLAX v2.2.15 (CLAMS) for metabolic cage studies, Image Lab 5.1 (Bio-Rad) for immunoquantification/Western blot; LAS AF version 2.6.0 build 7266 (Leica Microsystems CMS GmbH) and Imaris 9.1.2 (Bitplane AG) for acquisition of confocal images.
Data analysis	BLAST 5 and Espript 3.0 for protein sequence alignments, TANGO 2.3 for analysis of aggregation propensity, GraphPad Prism 8.3.0 and SigmaPlot v13 for preparation of figures and statistics; Discovery studio visualizer v9.1 for structural analysis and visualization; Image Lab 5.1 (Bio-Rad) for immunoquantification (Western blot); Fiji ImageJ 1.48o for Java 6 for image processing (particle size).

For manuscripts utilizing custom algorithms or software that are central to the research but not yet described in published literature, software must be made available to editors/reviewers. We strongly encourage code deposition in a community repository (e.g. GitHub). See the Nature Research guidelines for submitting code & software for further information.

Data

Policy information about availability of data

All manuscripts must include a data availability statement. This statement should provide the following information, where applicable:

- Accession codes, unique identifiers, or web links for publicly available datasets
- A list of figures that have associated raw data
- A description of any restrictions on data availability

The source data underlying Figures 1-6, Supplementary Figures 2c and 3-6, Tables 1 and 2 and Supplementary Tables 2 and 3 are provided as a Source Data file. The rest of the data that support Supplementary Figs. S7 and S8 within this paper, other findings and the mouse and derived material will be made available upon reasonable request to the correspondence author. Web-links for NCBI nucleotide sequences: Pah–mRNA NM_008777.3 https://www.ncbi.nlm.nih.gov/nuccore/NM_008777.3, Hsc70–mRNA NM_031165.4 https://www.ncbi.nlm.nih.gov/nuccore/NM_031165.4, Gch1–mRNA NM_008102.3 https://www.ncbi.nlm.nih.gov/nuccore/NM_008102.3, Hsf1–mRNA NM_008296.2 https://www.ncbi.nlm.nih.gov/nuccore/NM_008296.2, stub1–mRNA NM_019719.3 https://www.ncbi.nlm.nih.gov/nuccore/NM_019719.3, Gchfr –mRNA NM_177157.4 https://www.ncbi.nlm.nih.gov/nuccore/NM_177157.4, Dnajc12–mRNA NM_001253685.1 https://www.ncbi.nlm.nih.gov/nuccore/NM_001253685.1, p62-mRNA NM_001290769.1 https://www.ncbi.nlm.nih.gov/nuccore/

NM_001290769.1, Hsp70 –mRNA NM_001163434.1 https://www.ncbi.nlm.nih.gov/nuccore/NM_001163434.1, Ap-1–mRNA NM_001243043.1 https://www.ncbi.nlm.nih.gov/nuccore/NM_001243043.1, Gapdh-mRNA NM_008084.3 https://www.ncbi.nlm.nih.gov/nuccore/NM_008084.3
Web-link for PAH structure in complex with BH4 PDB 6HYC https://www.rcsb.org/structure/6HYC

Field-specific reporting

Please select the one below that is the best fit for your research. If you are not sure, read the appropriate sections before making your selection.

☒ Life sciences ☐ Behavioural & social sciences ☐ Ecological, evolutionary & environmental sciences

For a reference copy of the document with all sections, see nature.com/documents/nr-reporting-summary-flat.pdf

Life sciences study design

All studies must disclose on these points even when the disclosure is negative.

Sample size	The sample size for each experiment and parameter was calculated based on pilot studies. The experiments are set up to detect differences of 2-fold in mean value and in this manner an effect size of D=2.79 was computed. This parameter was subsequently used to calculate the sample size for a two-sided t-test comparison assuming a significance level of 5% and a power of 90%, providing a minimum of n=3-5 mice in each group. For most of the experiments additional mice were included to account for any unexpected event in order to maintain the statistical significance power of the experiment.
Data exclusions	No data were excluded from the analyses
Replication	Replication of the qPCR analyses. Replication of PAH activity and protein (by Western blot) in liver of 3 mouse genotyope groups. Replication of L-Phe challenge effect on blood L-Phe concentration in three individual experiments, confirmation was obtained in all experiments.
Randomization	Mice were allocated to their group based on their genotype, and we tried to obtain a 50:50 distribution of sex. The cages were randomly placed in the racks, and their order in the experiments were random. The mice in each cage were of the same sex, while the genotypes were mixed. This reduced the risk of cage effect.
Blinding	The cages were numbered randomly, and mice of different genotypes were housed in the same cage. This way the person performing the experiments was not aware of their genotype until the results were obtained and analysed.

Reporting for specific materials, systems and methods

We require information from authors about some types of materials, experimental systems and methods used in many studies. Here, indicate whether each material, system or method listed is relevant to your study. If you are not sure if a list item applies to your research, read the appropriate section before selecting a response.

Materials & experimental systems

n/a	Involved in the study	
☐	☒	Antibodies
☒	☐	Eukaryotic cell lines
☒	☐	Palaeontology
☐	☒	Animals and other organisms
☒	☐	Human research participants
☒	☐	Clinical data

Methods

n/a	Involved in the study	
☒	☐	ChIP-seq
☒	☐	Flow cytometry
☒	☐	MRI-based neuroimaging

Antibodies

Antibodies used

For Western blot:
Primary antibody mouse α-PAH (1:5000, Millipore-MAB5278), mouse α-ubiquitin (1:500, Thermo Fisher Scientific-131600); rabbit α-glyceraldehyde 3-phosphate dehydrogenase (GAPDH) (1:1000, Abcam-ab9485), secondary antibodies goat anti-mouse (GAM) (1:2500, Bio-Rad Laboratories) and goat anti-rabbit (GAR) (1:2500, Bio-Rad Laboratories), conjugated to horseradish peroxidase.

For Immunohistochemistry:
Antibodies rabbit anti-PAH (1:100, Abcam-ab178430), rabbit anti-PAH (1:800, Abcam-ab191415), goat α-Ubiquitin N-terminal (1:200, antibodies online-ABIN350072), rat anti-NUP98 (1:100, Abcam-ab50610), rat α-phospho-p62 (S403) (1:200, MBL-D343-3), goat anti-LC3B (1:100, Signalway antibody-C48312). Secondary antibodies donkey anti-rabbit IgG H&L (1:200; Alexafluor 488, Invitrogen-A21206), donkey anti-goat IgG H&L (1:400; Alexafluor 555, Abcam-ab150130), donkey anti-goat IgG (1:200; Cy3 conjugate, Millipore-AP180C), goat anti-rat IgG H&L (1:100; TRITC, Jackson immunoresearch-112-025-143), goat anti-rabbit IgG H&L (1:200; Alexafluor 647, Invitrogen-A21245), donkey anti-rat IgG H&L (1:200; Alexafluor 488, ThermoFisher Scientific-

A21208), goat anti-rabbit IgG H+L (1:200, HRP, Bio-Rad-1706515).

Validation

For Western blot:
1. α-PAH (Millipore-MAB5278)
- Species: Mouse monoclonal antibody
- Application: validated for WB, IP and IH
- Relevant citations: Cotton RGH et al., 1988 (PMID: 2461704)
- Data sheet on the manufacturer's website: https://www.merckmillipore.com/NO/en/product/Anti-Tryptophan-Hydroxylase-Tyrosine-Hydroxylase-Phenylalanine-Hydroxylase-Antibody-clone-PH8,MM_NF-MAB5278?ReferrerURL=https%3A%2F%2Fwww.labome.com%2Fproduct%2FEMD-Millipore%2FMAB5278.html&bd=1

Specific for phenylalanine hydroxylase (PAH) in hepatic lysates and sections.
2. α-ubiquitin (Thermo Fisher Scientific-131600)
- Species: Mouse monoclonal antibody
- Applications: WB, ELISA and IF
- Relevant citations: 79 references (see Manufacturer's website below; relevant publication Chen J et al., 2014, PDMI: 25395170)
- Data sheet on the manufacturer's website: https://www.thermofisher.com/antibody/product/Ubiquitin-Antibody-clone-Ubi-1-Monoclonal/13-1600

3. α-glyceraldehyde 3-phosphate dehydrogenase (GAPDH) (Abcam-ab9485)
- Species: Rabbit polyclonal antibody (loading control)
- Application: WB, IHC-P, IP, ELISA, IHC-Fr, ICC/IF, Flow Cyt
- Relevant citations: 1104 references; Relevant publications Lourenco MV et al., 2019 (PMID: 30617325), Hentilä J. et al., 2018 (PMID: 30713500).
- Data sheet on the manufacturer's website: https://www.abcam.com/gapdh-antibody-loading-control-ab9485.html

For Immunohistochemistry:
α-PAH (Abcam-ab178430):
-Species: Rabbit monoclonal antibody
-Application: WB, IHC-P, ICC/IF, Flow Cyt
--Data sheet on the manufacturer's website: https://www.abcam.com/pah-antibody-epr12380-ab178430.html
-The specificity of the PAH antibody was proven by an antigen pre-adsorption test (Supplementary Fig. 5).

2. α-NUP98 (Abcam-ab50610)
-Species: Rat monoclonal antibody
-Application (tested): WB, ICC/IF
-Relevant citations: 14 references
-Data sheet on the manufacturer's website: https://www.abcam.com/nup98-antibody-2h10-nuclear-pore-marker-ab50610.html

3. α-phospho-p62 (S403) (MBL-D343-3)
-Species: Rat monoclonal antibody
-Application: WB, IP, IHC
-Relevant citations: G Matsumoto et al., 2011 (PMID: 22017874)
-Data sheet on the manufacturer's website: https://www.mblintl.com/assets/D343-3-v2.pdf

4. α-Ubiquitin N-terminal (Antibodies-online-ABIN350072):
-Species: Goat polycolonal antibody
-Application: IHC, WB
--Data sheet on the manufacturer's website: https://www.antibodies-online.com/productsheets/ABIN350072.pdf

Animals and other organisms

Policy information about studies involving animals; ARRIVE guidelines recommended for reporting animal research

Laboratory animals

Homozygous knock-in PahR261Q/R261Q (Pah-R261Q) mice, in C57BL/6J genetic background. The PahR261Q/WT and PahWT/WT mice (siblings) from the breeding were utilized as control counterparts. In some of the experiments we also used Enu1 mice as control counterparts.
The age of the mice is indicated in the specific section for each assay, varying from 3 to 5 month old). Except for the study of body weight we did not find gender-associated variations for any other parameter or metabolite measured in this work, thus the mice groups for each experiment included evenly distributed males and females.

Wild animals

The study did not involve wild animals

Field-collected samples

The study did not involve samples collected from the field.

Ethics oversight

The animal studies were approved by the Norwegian Food Safety Authority and performed at
the Laboratory Animal Facility, University of Bergen, according to the guidelines and

standards from the Regulation on the use of animals in research of this institution.

Note that full information on the approval of the study protocol must also be provided in the manuscript.
